# Intrinsic challenges in ancient microbiome reconstruction using 16S rRNA gene amplification

**DOI:** 10.1038/srep16498

**Published:** 2015-11-13

**Authors:** Kirsten A. Ziesemer, Allison E. Mann, Krithivasan Sankaranarayanan, Hannes Schroeder, Andrew T. Ozga, Bernd W. Brandt, Egija Zaura, Andrea Waters-Rist, Menno Hoogland, Domingo C. Salazar-García, Mark Aldenderfer, Camilla Speller, Jessica Hendy, Darlene A. Weston, Sandy J. MacDonald, Gavin H. Thomas, Matthew J. Collins, Cecil M. Lewis, Corinne Hofman, Christina Warinner

**Affiliations:** 1 Faculty of Archaeology, Leiden University, Einsteinweg 2, 2333 CC, Leiden, the Netherlands; 2 Department of Anthropology, University of Oklahoma, Norman, OK, USA; 3 Center for Geogenetics, University of Copenhagen, Denmark; 4 Department of Preventive Dentistry, Academic Center for Dentistry Amsterdam, University of Amsterdam and VU University Amsterdam, the Netherlands; 5 Department of Anthropology, University of Cape Town, South Africa; 6 Departament de Prehistòria i Arqueologia, Universitat de València, Spain; 7 Department of Human Evolution, Max-Planck Institute for Evolutionary Anthropology, Leipzig, Germany; 8 School of Social Sciences, Humanities, and Arts, University of California, Merced, USA; 9 Department of Archaeology, University of York, York, UK; 10 Department of Anthropology, University of British Columbia, Vancouver, Canada; 11 Department of Biology, University of York, York, UK

## Abstract

To date, characterization of ancient oral (dental calculus) and gut (coprolite) microbiota has been primarily accomplished through a metataxonomic approach involving targeted amplification of one or more variable regions in the 16S rRNA gene. Specifically, the V3 region (*E. coli* 341–534) of this gene has been suggested as an excellent candidate for ancient DNA amplification and microbial community reconstruction. However, in practice this metataxonomic approach often produces highly skewed taxonomic frequency data. In this study, we use non-targeted (shotgun metagenomics) sequencing methods to better understand skewed microbial profiles observed in four ancient dental calculus specimens previously analyzed by amplicon sequencing. Through comparisons of microbial taxonomic counts from paired amplicon (V3 U341F/534R) and shotgun sequencing datasets, we demonstrate that extensive length polymorphisms in the V3 region are a consistent and major cause of differential amplification leading to taxonomic bias in ancient microbiome reconstructions based on amplicon sequencing. We conclude that systematic amplification bias confounds attempts to accurately reconstruct microbiome taxonomic profiles from 16S rRNA V3 amplicon data generated using universal primers. Because *in silico* analysis indicates that alternative 16S rRNA hypervariable regions will present similar challenges, we advocate for the use of a shotgun metagenomics approach in ancient microbiome reconstructions.

The human body harbors an astounding number of microorganisms, collectively known as the *human microbiome*^1^. The number of these microorganisms (approx. 100 trillion) is estimated to exceed that of the human cells in our bodies (approx. 10 trillion) by an order of magnitude^2^, and the number of microbial genes in our microbiome (approx. 3,300,000) has been shown to outnumber our own (approx. 22,000) by a factor of more than 150^3^. These microbial genes encode a wide range of biological functions including those related to host nutrient acquisition, metabolism and immunity^4^,^5^,^6^,^7^,^8^. Moreover, recent comparative studies with non-human primates suggest rapid changes in human microbiota occurred during human evolution^9^. Consequently, there is tremendous interest in characterizing the evolutionary ecology of the human microbiome through the direct investigation and comparative analysis of both modern and ancient forms^10^,^11^,^12^.

Recent studies of microbiome variation in humans^13^,^14^,^15^,^16^,^17^ and non-human primates^9^,^18^,^19^,^20^,^21^ have primarily characterized host-associated microorganisms using an amplicon metataxonomic approach^22^, in which a targeted variable region of the 16S rRNA gene is amplified by polymerase chain reaction (PCR), deep sequenced using Next Generation Sequencing (NGS) technology, and compared to a reference database of 16S rRNA gene sequences. The 16S rRNA gene encodes the small subunit of prokaryotic ribosomal RNA and contains nine hypervariable regions (V1-V9) separated by ten highly conserved regions^23^. It is the most widely used gene in microbial metataxonomic analysis, in part because the 16S rRNA gene is sufficiently conserved across members of the paraphyletic prokaryotic domains Bacteria and Archaea to allow the design of “universal” primers for microbial PCR amplification, yet also sufficiently variable to allow full 16S rRNA sequences to be classified at an approximate species level. The 16S rRNA gene is among the most studied and best characterized genes among prokaryotes, and more than 100,000 full 16S rRNA sequences are available for microbial taxa in publicly accessible databases such as RDP^24^, SILVA^25^, and Greengenes^26^.

While it is possible to amplify and sequence the entire 16S rRNA gene (approx. 1540 bp) using conventional cloning and Sanger sequencing, this approach is impractical for high-throughput studies of microbial communities. Alternatively, a shorter target comprising one or more hypervariable regions may be amplified and sequenced on an NGS platform, allowing for high coverage characterization of hundreds of samples simultaneously. For example, the Earth Microbiome Project^27^ has developed a standardized and widely used NGS assay for microbiome characterization that targets the V4 region of the 16S rRNA gene using the universal primers 515F/806R^28^. However, the relatively long length of the V4 region (approx. 292 bp, primer inclusive) makes it impractical for ancient DNA (aDNA) studies given that aDNA is known to be highly fragmented and rarely exceeds 200 bp in length^29^.

Alternatively, ancient microbial studies have targeted other hypervariable regions in the 16S rRNA gene, including V1^30^,^31^,^32^,^33^, V1-V2^32^,^34^, V3^30^,^32^,^33^,^35^,^36^, V1-V3^32^, V5^36^, and V6^30^,^36^. Following both *in silico* and *in vitro* testing of multiple 16S rRNA gene variable region primer pairs, two studies concluded that the V3 region was an optimal target for ancient microbial studies^30^,^36^ because: (1) it is relatively short (approximately 100 bp shorter than the V4 region); (2) it exhibits high sequence variation, resulting in good taxonomic discrimination; and (3) it is less subject to primer taxonomic bias than other primer pairs when compared to data generated using non-targeted (shotgun) whole metagenome sequencing.

Despite these characteristics, we have recently observed that for many ancient microbiome samples, the microbial community profiles reconstructed from targeted sequencing of the 16S rRNA gene V3 region do not conform to biological expectations and show systematic taxonomic biases, such as exceptionally high frequencies of the human-associated archaeon *Methanobrevibacter*, that cannot be explained by exogenous contamination. Although 16S rRNA amplicon sequencing has been the primary means of characterizing ancient microbiome samples since 1998^30^,^31^,^32^,^33^,^34^,^35^,^36^,^37^, no study has yet systematically investigated the effect of aDNA fragmentation on the fidelity of amplicon-based ancient microbiome reconstructions. Because of this, it is unclear how to interpret reported differences observed between modern and ancient microbial communities^30^.

To address this problem, we conducted a series of *in silico* and *in vitro* experiments to explore the role of inherent structural variation in the 16S rRNA gene on downstream microbiome reconstruction from archaeological specimens. First, we present and describe microbiome profiles generated by targeted amplicon sequencing of the V3 region for a large collection of archaeological dental calculus specimens (n = 107). Then, we used non-targeted paired-end shotgun metagenome sequencing to empirically determine the length distribution of aDNA fragments in a subset of four specimens from diverse geographic and temporal contexts. Next, using sequences in the SILVA SSU 111 database, we investigated the V3 region of the 16S rRNA gene for primer bias, amplicon length, and variation in amplicon length, and compared microbiome profiles between amplicon and shotgun metagenome datasets for the four archaeological dental calculus specimens. We then estimated the probability of a nucleotide being damaged (λ) assuming a random degradation model and simulated the effect of this damage on taxonomic profiles generated from a hypothetical oral microbiome at different thermal ages. We show that, assuming random degradation, longer targets will be underrepresented in thermally older samples, leading to a perceived shift in V3 target composition. We conclude that extensive length polymorphisms in the V3 region are a major cause of amplification dropout and taxonomic bias in ancient microbiome reconstructions generated by amplicon sequencing. Overamplification of archaeal taxa and altered microbial diversity estimates are predictable artifacts observed in poorly preserved (highly fragmented) but relatively uncontaminated aDNA samples. Finally, we analyzed 16S rRNA gene sequences in taxonomically diverse microorganisms in the SILVA SSU 111 16S rRNA database and evaluated the other hypervariable regions on a variety of quality metrics, including predicted primer amplification bias, median amplicon length, and variation in amplicon length. We demonstrate that although amplicon-based 16S rRNA gene sequencing may be a useful high-throughput screening tool for qualitative characterization of the preservation and contamination burden of ancient microbiome samples, it cannot be used to reliably reconstruct quantitative information regarding microbial diversity or taxonomic frequency in ancient microbial communities.

## Results

### Taxonomic analysis of 16S rRNA V3 amplicon sequence data generated from dental calculus

**Figure 1:**
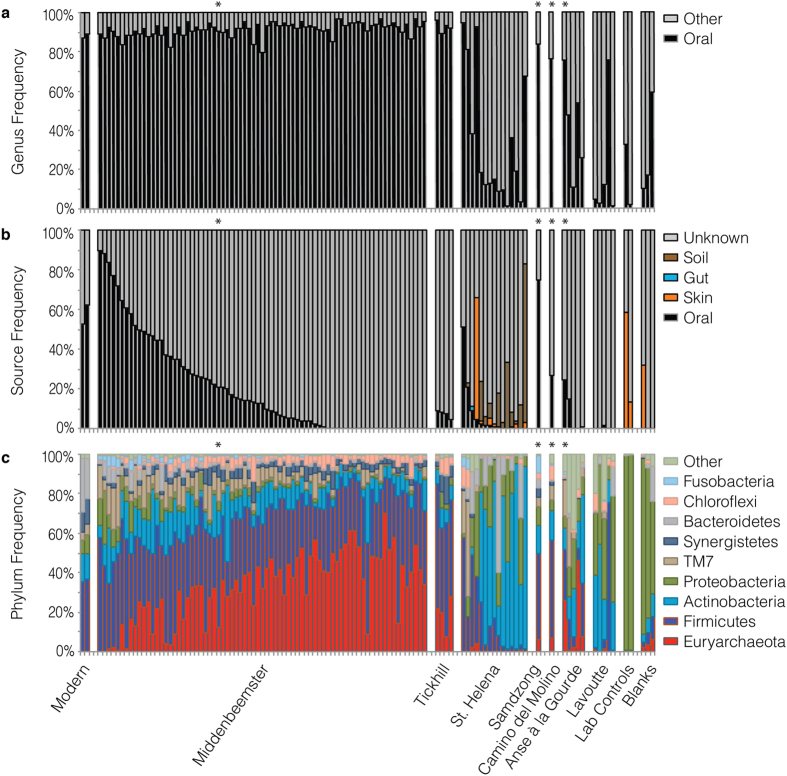
Unusual microbiome profiles observed in 16S rRNA gene V3 amplicon data from archaeological dental calculus. Relative abundance charts summarizing: (**a**) Frequency of oral-associated genera in dental calculus and control samples. The Dutch, UK, Nepalese, and Spanish calculus samples show a greater proportion of oral-associated genera compared to the St. Helena and pre-Columbian Caribbean samples. (**b**) Contribution of oral, gut, and environmental sources to microbiome composition estimated by Bayesian source tracking. The oral microbiome (saliva, supragingival plaque, subgingival plaque) is a major source (>50%) in only a small proportion of archaeological dental calculus samples (10%), and an oral source is not indicated for more than a quarter of samples (26%). Laboratory controls (osteologist hands and osteology lab bench surfaces) and extraction blanks are largely consistent with a skin microbiome source and unassigned contaminants. (**c**) Frequency of microbial phyla inferred from V3 amplicon sequencing. The taxonomic profile reveals an unusual and non-biological pattern of exceptionally high Euryarchaeota levels in the Dutch, UK, and some Caribbean dental calculus samples. All Euryarchaeota OTUs are assigned to the genus *Methanobrevibacter*, the only prevalent genus of Archaea in the oral cavity. *Methanobrevibacter* is typically found at low frequencies (<0.5%) in healthy human dental plaque^79^, but in archaeological samples it may reach frequencies >60%, as seen here. Starred samples (*) were also analyzed using shotgun metagenome sequencing. Sites are ordered from left to right by increasing thermal age (see Table 1). Figure data is available in Supplementary Data 1.

To investigate taxonomic profiles generated by amplicon sequencing in temporally and geographically diverse archaeological dental calculus specimens, we selected and analyzed samples (n = 107) from five sites: Middenbeemster, the Netherlands (n = 76); Rupert’s Valley, St. Helena (n = 15); Anse à la Gourde, Guadeloupe (Caribbean) (n = 5); Lavoutte, St. Lucia (Caribbean) (n = 5); Tickhill, Yorkshire, UK (n = 4); Samdzong, Nepal (n = 1); and Camino del Molino, Spain (n = 1) (Table 1; Supplementary Table 1). A larger number of samples were profiled from the Dutch Middenbeemster cemetery in order to examine oral microbiome variation within a single site. Following deep sequencing of 16S rRNA gene V3 amplicons on an Illumina MiSeq platform, we assigned sequences to Operational Taxonomic Units (OTUs) at 97% similarity using the closed-reference OTU protocol implemented in QIIME^38^,^39^, and the Greengenes 13.8 database^26^ as a reference (see Methods). We then analyzed the resulting taxonomic data using three complementary approaches (Fig. 1): (a) by scoring the assigned genera to the categories of “Oral” or “Other”, as inferred by the presence or absence of each genus in the Human Oral Microbiome Database (HOMD)^40^ and tabulating the resulting frequency data; (b) by proportionally assigning the sample data to human microbiome and environmental sources using the Bayesian microbial source tracking program SourceTracker^41^; and (c) by directly examining the phylum frequency data. A modern dental calculus sample (analyzed in duplicate), two laboratory controls (osteologist hand swab and osteology lab bench swab), and three extraction blanks (non-template negative extraction controls) were also analyzed in parallel and are provided for comparison. Median sequencing depths for the dental calculus samples was high: modern (96,157), Middenbeemster (85,478), Tickhill (68,170), St. Helena (10,941), Samdzong (68,253), Camino del Molino (90,416), Anse à la Gourde (41,271), and Lavoutte (38,229). Lower sequencing depths were achieved for the laboratory controls, in part because of their lower starting biomass: osteologist hand swabs (20,368 read pairs), osteology lab bench tops (4,617 read pairs). No amplification was observed for the extraction or PCR blanks, but they were nevertheless prepared into libraries. Accordingly, sequencing depth for the three blanks was very low: 17, 190, and 316 read pairs.

The results of the first approach reveal a high proportion of oral-associated genera in the Middenbeemster (median 92%) and Tickhill (median 92%) dental calculus samples, comparable to modern dental calculus (median 88%) (Fig. 1a). The non-oral taxa in the modern dental calculus sample consist almost entirely of *Paludibacter*, a genus of commensal plant bacteria that may be of dietary origin. The Samdzong and Camino del Molino samples likewise exhibit a high proportion of oral-associated genera (84% and 76%, respectively), despite their greater age. By contrast, the Caribbean and St. Helena samples are highly variable in composition, and only 3 of the 10 Caribbean samples and 4 of the 15 St. Helena samples contain oral-associated genera at a frequency of >50%. The remaining samples are dominated by soil and environmental taxa, including members of the orders Actinomycetales, Acidimicrobiales, Nitrospirales, Rhizobiales, Rhodospirillales, Solirubrobacterales, and Xanthomonadales. This pattern is consistent with a high degree of specimen degradation and exogenous soil contamination, a pattern not unexpected for specimens obtained from a tropical environment with a high thermal age^42^,^43^. The laboratory control samples are dominated by a narrow range of nasal and skin-associated taxa, notably Oxalobacteriaceae and Moraxellaceae, which comprise >80% of the sample. *Acinetobacter* and *Pseudomonas*, two genera associated with both the skin and oral microbiome, were also observed. The extraction blanks include a diverse range of soil, skin, and oral microbe sequences, all found at very low absolute abundance (17–316 sequences).

Applying Bayesian source tracking^41^ to the same dataset, a stark pattern emerges. Although the SourceTracker results broadly confirm several of the above observations – that the Caribbean dental calculus preservation is generally poor, that the Samdzong and Camino del Molino calculus preservation is better, and that skin microbes have contributed to the composition of the control samples – the Middenbeemster samples exhibit a gradient of oral microbiome contribution that differs sharply from that inferred from constituent genera alone (Fig. 1b). Interestingly, this gradient is inversely correlated with the proportion of reads assigned to the archaeal phylum Euryarchaeota (Spearman’s rho = −0.30, *p* < 0.001) (Fig. 1c).

Examining the dental calculus OTU assignments more closely, nearly all of the Euryarchaota sequences (>99.8%) can be assigned to the genus *Methanobrevibacter*. In the oral cavity, *Methanobrevibacter* is a genus with low abundance (<0.01% in our modern control), represented almost exclusively by the taxon *M. oralis*. Thus, the exceptionally high and variable *Methanobrevibacter* frequencies observed within the archaeological dental calculus, and especially within the Middenbeemster samples (median 33.8%), are unlikely to reflect a biological pattern. Likewise, within Chloroflexi, the vast majority of sequences (>98.5%) can be assigned to a single OTU within Anaerolinaceae. This bacterial family, which is present but not well characterized within the human oral cavity, is also typically found at low abundance (<0.01% in our modern control), but is found at a median frequency of 3% in our archaeological samples.

These observed patterns are difficult to explain on the basis of present evidence. Contamination is an unlikely cause of the taxonomic skew in the Middenbeemster samples, in part because the taxa that are overrepresented are members of the oral microbiome. Alternatively, postmortem microbial overgrowth or biased amplification due to taphonomic alteration are two additional possibilities. To test whether either or both of these factors could be the cause of the taxonomic skew, we selected and analyzed a subset of archaeological human dental calculus samples using shotgun metagenome sequencing.

### Shotgun characterization of dental calculus samples

From our initial pool of 107 dental calculus samples, we selected four samples (Table 1) for further analysis using non-targeted shotgun metagenomics: 454C (Middenbeemster), F1948C (Anse à la Gourde), 37C (Samdzong), and 214C (Camino del Molino). The shotgun and amplicon libraries were built from the same original set of aDNA extracts, and the samples were selected from different sites to ensure that the results would not be biased by a specific geographic location or temporal period.

Overall, DNA yields obtained from these ancient dental calculus samples were high (24.7–60.5 ng/mg), as is now known to be typical for this mineralized biofilm^10^,^36^. The extracts were built into Illumina libraries and sequenced on an Illumina HiSeq 2000 NGS platform run in 2 × 100 PE mode. After filtering 16S rRNA gene sequences from the metagenomics dataset, we assigned taxonomy following the methods described above and compared the results to the amplicon data. Paired comparisons reveal that median *Methanobrevibacter* and *Anaerolineae G-1* levels are consistently lower in the shotgun data (1.6%, 1.3%, respectively) than the amplicon data (9.4%, 2.7%, respectively) for the same samples. In some cases this difference is extreme, as observed for F1948C, where the frequency of *Methanobrevibacter* is 26.1% in the amplicon dataset and 2.2% in the shotgun dataset. Thus, postmortem microbial overgrowth does not explain the taxonomic skew observed in the amplicon data. Next, we considered DNA preservation and possible amplification biases.

### DNA fragmentation in ancient dental calculus

**Figure 2:**
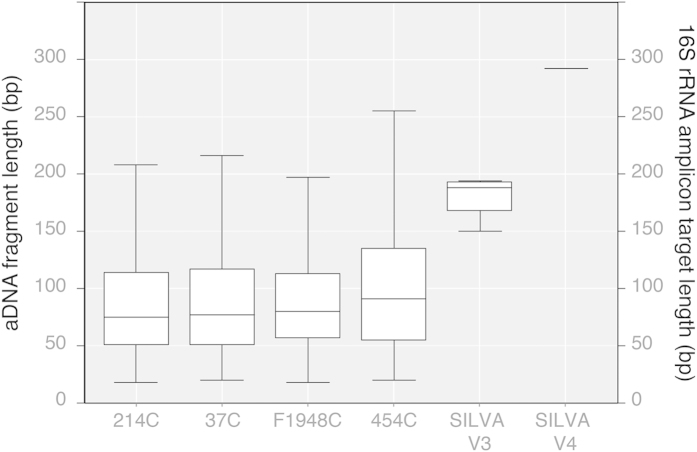
Length distribution box plots of aDNA extracted from archaeological dental calculus and calculated V3 and V4 16S rRNA amplicon lengths for microbes in the SILVA SSU 111 database. As expected for aDNA, the genetic material within dental calculus is highly fragmented to median lengths <100 bp: 214C, 75 bp; 37C, 77 bp; F1948C, 80 bp; 454C, 91 bp. This is significantly shorter than the median lengths of the 16S rRNA V3 (183 bp) and V4 (292 bp) amplicon targets. The number of read pairs comprising each box plot are as follows: 214C, 20,355; 37C, 17,962; F1948C, 26,517; 454C, 15,736; SILVA V3, 651,163; SILVA V4, 649,660.

In general, aDNA is known to be highly degraded and fragmented^29^,^42^,^44^. However, the fragmentation of aDNA within dental calculus is not well explored. To investigate the degree of DNA fragmentation in our samples, we filtered the paired-end reads for 16S rRNA gene sequences by aligning them to the SILVA SSU 111 database. Fragment length distribution for this gene within each sample was then estimated using Picard-tools^45^. Median DNA fragment lengths were relatively short (75–91 bp), as expected for authentic aDNA. Median fragment length was greatest in the youngest sample (454C) and was observed to decrease with the age of the sample (Fig. 2). DNA fragmentation and nucleotide misincorporation profiles were also consistent with authentic aDNA, and cytosine deamination was observed to significantly increase with sample thermal age (Kruskal-Wallis, *p* < 0.01; Supplementary Figure 1).

In addition to age effects, it has also been hypothesized that that cell wall composition may impact DNA fragmentation and damage patterns in archaeological samples^30^, and such effects have been reported for ancient *Mycobacterium leprae* recovered from bone and dentine^46^. To test this hypothesis in our dental calculus samples, we compared aDNA sequences from two Gram-positive (*Streptococcus gordonii* and *Propionibacterium propionicum*) and two Gram-negative (*Lautropia mirabilis* and *Porphyromonas gingivalis*) oral bacteria. We found that differences in DNA fragment length and cytosine deamination rates are driven by specific taxa rather than by cell wall composition (Supplementary Figure 2). For example, *P. gingivalis* was observed to have shorter median DNA fragment lengths across all samples compared to the other three taxa (Kruskal-Wallis, *p* = 0.04), while *S. gordonii* exhibited the lowest median cytosine deamination rate. This suggests that other, as yet unknown, factors may contribute to differential preservation of microbial DNA within archaeological dental calculus.

For all ancient samples, the median DNA fragment length was found to be substantially shorter than the median required template length for V3 U341F/534R amplification (183 bp), and the lengths of the V3 U341F/534R amplicons fall within the 4^th^ quartile of the dental calculus aDNA distributions, suggesting that amplification is possible from only a minor proportion of the total ancient 16S rRNA gene template molecules (214C, 2.9–7.5%; 37C, 3.1–8.2%; F1948C, 2.0–6.6%; 454C, 6.6–13.5%; ranges represent percentage of fragments available for upper and lower limits of V3 region lengths, respectively). Because the number of aDNA fragments that are sufficiently long for targeted V3 U341F/534R PCR amplification is both low and variable across samples, taxonomic dropout and stochastic effects are expected because not all molecules are equally accessible to dual primer hybridization. The extent of this amplification bias (differential PCR amplification) is expected to scale with sample complexity. As highly complex systems, microbiomes are particularly vulnerable to this bias^47^. However, stochastic processes alone cannot account for the consistent taxonomic shifts, such as the overrepresentation of Euryarchaeota observed in our ancient dental calculus samples. One possible explanation for consistent taxonomic shifts resulting from differential PCR amplification is variation in PCR amplicon length, as is evident for V3 U341F/534R (Fig. 2). To test whether inherent amplification biases could be the cause of the taxonomic skew, we examined the V3 region of the 16S rRNA gene in greater detail.

### Characteristics of 16S rRNA V3 region

#### Taxonomic coverage

The V3 region of the 16S rRNA gene (*E. coli* position 341–534 bp) has been extensively documented for its ability to distinguish microbial genera through both sequencing and DGGE based studies. To better understand taxonomic coverage exhibited by the V3 region, we used PrimerProspector^48^ to evaluate *in silico* amplification of 16S rRNA sequences from the SILVA database using the V3 U341F/534R primer pair. Overall, the primer pair showed approx. 98% recovery for bacterial sequences and approx. 91% recovery for archaeal sequences, distributed over 46 major phyla each with over 100 representative sequences in the database. When limited to phyla commonly found in the human oral cavity, this primer pair has a >97% recovery rate with the exception of Euryarchaeota and Chlamydiae, where the numbers fall to 91% and 71% respectively. However, these primers do amplify *Methanobrevibacter oralis* and *Chlamydophila pneumoniae*, the only known members of Euryarchaeota and Chlamydiae, respectively, in the oral cavity. Thus, the taxonomic skew observed in the V3 amplicon data for Middenbeemster, Samdzong, and Camino del Molino samples are unlikely to be attributed to predicted taxonomic biases in primer binding for the U341F/534R primer pair.

#### Length polymorphisms in the V3 region

**Figure 3:**
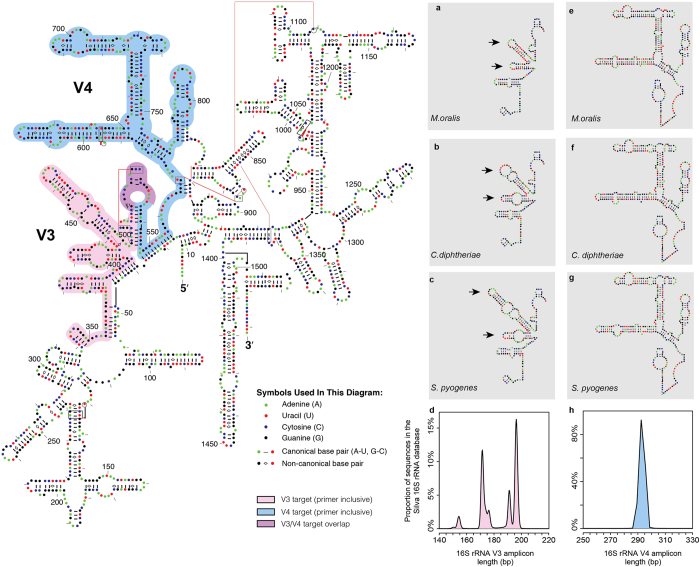
Simplified 16S small subunit ribosomal RNA secondary structure. Secondary structure of *Escherichia coli* (J01695) 16S rRNA (main panel). Amplicon targets (primer inclusive) for the third (V3, 341F/529R) and fourth (V4, 515F/806R) variable regions are highlighted in pink and blue, respectively. Overlapping V3/V4 target sequences are highlighted in purple. Although widely used in ecological studies, the V4 region is impractical for aDNA research because of its long length (approx. 292 bp, primer inclusive). The V3 region is considerably shorter, but comparative sequence analysis (**a–d**) reveals that the V3 region exhibits extensive length polymorphisms (arrows) in archaeal (e.g., *Methanobrevibacter oralis*) and bacterial (e.g., *Corynebacterium diphtheria*, *Streptococcus pyogenes*) taxa, with predicted V3 amplicon lengths ranging from 150–194 bp when queried against the SILVA SSU 111 16S rRNA database (**d**). By contrast, the V4 region is relatively length invariant (**e–h**), ranging from 290–295 bp (**h**). 16S rRNA secondary structure has been adapted from Comparative RNA Web Site and Project^78^.

Next, we examined amplicon distribution profiles for the V3 U341F/534R primers. While the V3 region is often described as being <200 bp in length (*E. coli*), more specifically, we observe a multimodal distribution in amplicon lengths, differing by as much as 44 bp (150–194 bp). In comparison, the commonly used V4 primer pair 515F/806R has a median amplicon length of 292 bp, with a range of 290–295 bp. Because the 16S rRNA gene encodes the small subunit of prokaryotic ribosomal RNA, its sequence reflects the properties of rRNA secondary folding structure (Fig. 3). Here we have highlighted the parts of the 16S rRNA corresponding to the V3 U341F/534R (pink) and V4 515F/806R (blue) gene amplicon targets, which overlap in the 534R and 515F primer binding sites (purple). Within these regions variation occurs through both SNPs and Insertion/Deletions (indels), with the V3 region having both types, and the V4 region having primarily SNPs. Specifically, the V3 region contains two stem-loop structures exhibiting length polymorphisms that differ across taxonomic clades (Fig. 3a–c, arrows). The shortest stem-loop structures are found in archaea, such as *Methanobrevibacter oralis* (Fig. 3a), while bacteria exhibit high length variability within these structures, as observed for *Corynebacterium diphtheria* (Fig. 3b) and *Streptococcus pyogenes* (Fig. 3c). In contrast, the V4 region is relatively length invariant and exhibits no major structural variation in stem loop structures for these same taxa (Fig. 3e–g).

**Figure 4:**
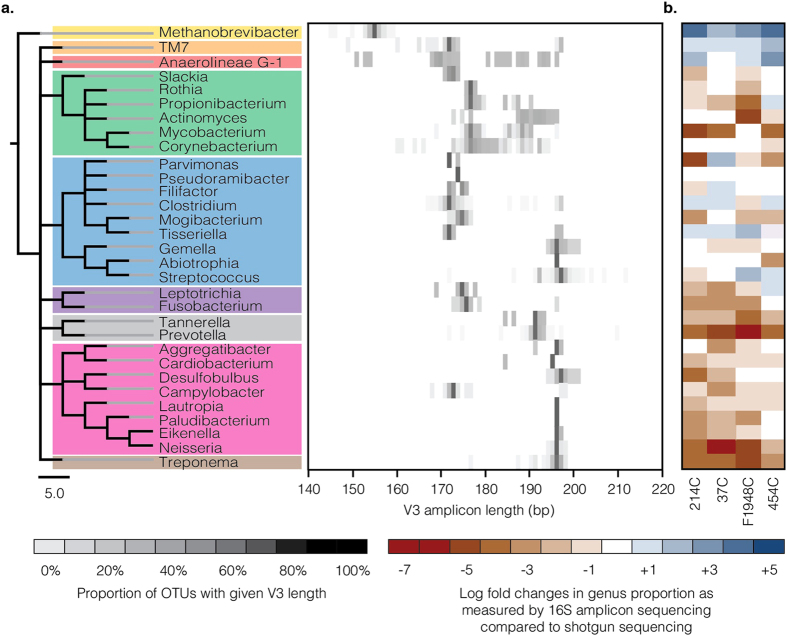
Heatmap of 16S rRNA V3 amplicon lengths reveals high variability but broad taxonomic patterns. (**a**) 16S rRNA V3 sequence data was analyzed *in silico* for 36,634 OTUs belonging to 31 representative oral microbiome genera from 9 major microbial phyla: Euryarchaeota (yellow), TM7 (orange), Chlorflexi (red), Actinobacteria (green), Firmicutes (blue), Fusobacteria (purple), Bacteroidetes (gray), Proteobacteria (pink), Spirochaetes (brown). (**b**) Log fold changes in genus frequency within archaeological dental calculus when comparing data generated by targeted V3 U341F/534R amplicon sequencing to non-targeted shotgun metagenomics data. *Methanobrevibacter*, *Anaerolinea*, and TM7, which have very short predicted V3 U341F/534R amplicon lengths, strongly overamplify compared to frequency data obtained from non-targeted shotgun metagenomic sequencing. Most other taxa underamplify, especially genera with very long predicted V3 U341F/534R amplicon lengths, such as *Treponema* and members of Proteobacteria, and Bacteroidetes.

To provide greater resolution of taxonomic associations of the V3 region length polymorphisms, we systematically analyzed predicted V3 U341F/534R amplicon lengths at the phylum and genus levels using PrimerProspector and the SILVA SSU 111 database. We selected 31 representative oral genera from nine phyla for analysis and plotted a heatmap of the distribution of the predicted V3 U341F/534R amplicon lengths for all OTUs assigned to these genera (Fig. 4a). Clear taxonomic associations are observed with respect to predicted amplicon length. As previously noted, the archaeon *Methanobrevibacter* has the shortest predicted amplicon length (median 151 bp), followed by the bacteria *Anaerolineae G-1* (median 169 bp) and the bacterial candidate phylum TM7 (median 169 bp). *Treponema* and Proteobacteria have among the longest predicted amplicon length (median >191 bp, except *Campylobacter*). The length of the V3 region varies widely among Actinobacteria, even within the same genus, and several phyla, especially Actinobacteria, Firmicutes, and Bacteroidetes, contain genera with multimodal length distributions. To confirm that these results are not a database artifact, we repeated this exercise using the RDP, NCBI, and Greengenes databases, and found comparable results (Supplementary Figure 3).

Interestingly, unusually high levels of *Methanobrevibacter* are a common feature of the taxonomic skew observed in amplicon based characterization of dental calculus. This suggests that length differences in the V3 region play a role in this taxonomic skew, with taxa having shorter V3 regions showing better amplification than those with longer V3 regions.

### Comparison of taxonomic profiles generated by targeted amplicon and shotgun metagenome sequencing

**Figure 5:**
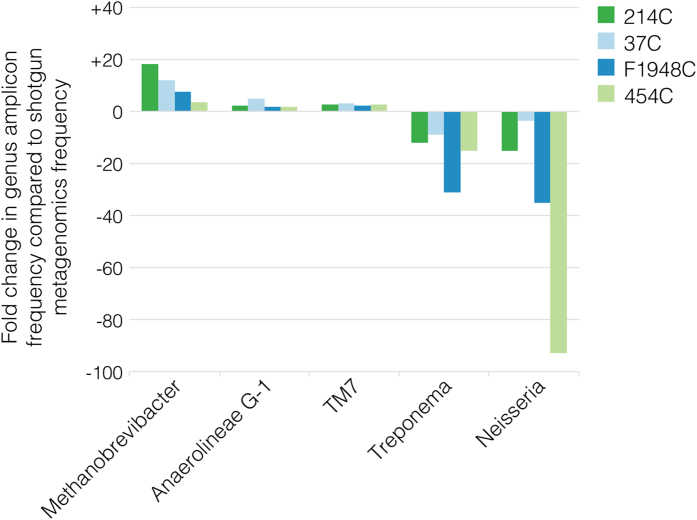
Fold changes in taxon frequency between 16S rRNA V3 U341F/534R amplicon and shotgun metagenome data. Genera with relatively short median amplicon lengths (*Methanobrevibacter*, Anaerolineae *G-1*, TM7) are overrepresented in the 16S rRNA V3 U341F/534R amplicon dataset, while genera with relatively long median amplicon lengths (*Treponema*, *Neisseria*) are strongly underrepresented.

Given the highly fragmented nature of aDNA, length polymorphisms in the V3 region of the 16S rRNA gene may lead to differential PCR amplification, specifically overamplification of taxa with very short V3 regions and underamplification or taxonomic dropout of taxa with very long V3 regions. To test this hypothesis, we generated and compared taxonomic frequency data from paired V3 U341F/534R amplicon libraries and shotgun metagenome libraries built from the four archaeological dental calculus samples described above. Rarefaction analyses show plateauing for both the amplicon and shotgun metagenome datasets indicating sufficient sampling for analysis (Supplementary Figure 4). We found that the amplicon datasets exhibit an excess of taxa with very short V3 sequences (e.g., *Methanobrevibacter*, *Anaerolineae G-1,* and TM7), and a deficiency of taxa with very long V3 sequences (e.g., *Treponema*, *Neisseria*, and *Prevotella*) (Fig. 4b). Hence, genera with shorter median fragment lengths, such as *Methanobrevibacter*, are strongly overrepresented using an amplicon sequencing approach, whereas genera with longer median fragment lengths, such as *Treponema*, are strongly underrepresented, with other taxa exhibiting intermediate frequency changes (Fig. 5). In addition to the V3 region length, the magnitude of this bias is likely to depend on both the degree of postmortem aDNA degradation and the original relative abundance of the DNA template in the sample.

### Predicted V3 U341F/534R amplicon lengths of specific oral microbiome taxa of interest

Overall, our comparative data indicate that V3 U341F/534R amplification results in a highly skewed taxonomic profile when applied to archaeological microbiome samples. Nevertheless, because amplicon sequencing can be scaled for high-throughput analysis and is more economical on a per sample basis than shotgun metagenome sequencing, it may still have value as a sample screening tool prior to shotgun metagenome analysis. In this case, it is important to know which taxa are likely to over- or underamplify, or perhaps dropout altogether. In Table 2 we provide the predicted V3 U341F/534R amplicon lengths for a range of oral microbiome taxa of interest, including prominent commensals and pathogens implicated in caries formation, periodontal disease, respiratory illness, and a variety of acute and chronic systemic diseases. It is worth noting that most pathogenic taxa have very long V3 regions and hence are likely to be underrepresented or absent from V3 U341F/534R amplicon datasets generated from archaeological material. This has important implications for high-throughput sample screening using this approach because low abundance pathogens, such as *Mycobacterium tuberculosis*, are likely to drop out.

Finally, because of the close evolutionary relationship between chloroplasts and cyanobacteria^49^, the chloroplast 16S rRNA gene is also amplified by the V3 U341F/534R primers (170 bp amplicon). Chloroplast sequences have been previously reported in modern dental plaque^50^, and we have observed them at variable, but generally very low, frequencies in archaeological dental calculus. Although such sequences indicate the presence of chloroplast DNA, and thus possible dietary components, sequence variation within the V3 U341F/534R amplicon is insufficient to resolve taxonomy below the level of Streptophyta, an unranked plant clade that includes all land plants and some green algae.

### Theoretical modeling of the effect of thermal age on oral microbiome taxonomic frequencies

**Figure 6:**
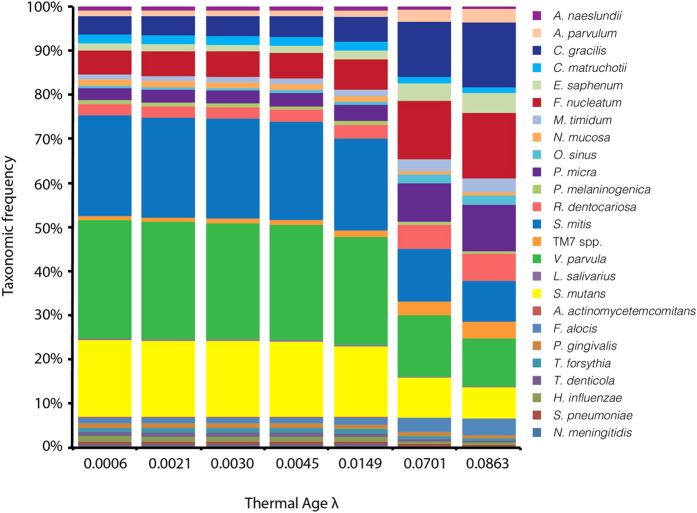
Predicted effect of thermal age on reconstructed taxonomic frequencies of selected oral bacteria from V3 U341F/534R amplicon data. Using a random DNA degradation model, the relative abundance of taxa presented in Table 2 is modeled at different thermal ages, corresponding to the thermal ages of the archaeological sites in this study. The probability of chain scission (λ)^80^ is estimated using temperatures estimated using previously published kinetic parameters^81^. Starting taxonomic frequencies were taken from mean HOMD values, and taxa with a frequency of <0.01% in the HOMD are not shown. Together, the taxa shown account for 25% of the HOMD human oral microbiome.

To explore the diachronic effects of DNA degradation on a single sample, we used thermal age calculations^42^,^43^ to model predicted taxonomic skew in a hypothetical oral microbiome sample evaluated at multiple thermal ages. We estimated the probability of a nucleotide being damaged (λ) resulting in chain scission assuming a random degradation model for each of the archaeological sites in this study (Table 1). Using this probability term, we then explored the impact that temperature history would have on the relative survival of the V3 regions of the taxa presented in Table 2 for a hypothetical oral microbiome sample averaged from all entries in the HOMD. In this model, the probability of a DNA fragment of size *x* or greater being present is given by e^−λ*x*^. As such, samples of greater thermal age will exhibit greater fragmentation, and within a sample, longer templates have a higher probability of experiencing strand breakage than shorter templates. By modeling the amplification success of DNA templates within a hypothetical microbiome sample at multiple thermal ages, we observe a clear pattern whereby specific taxa systematically increase or decrease in frequency simply as a function of aDNA degradation (Fig. 6).

Overall, these results confirm the biases inherent to aDNA microbial community characterization using V3 region primers. Next, to evaluate the suitability of the other variable regions for aDNA community analysis, we performed extensive *in silico* analyses to examine taxonomic and length biases in these regions.

### *In silico* evaluation of 16S rRNA gene and universal primers

When selecting universal 16S rRNA primers, two characteristics are paramount: high amplicon taxonomic resolution and high amplicon taxonomic coverage. With respect to primers that will be applied to aDNA, short amplicon length is also critical given the known fragmented and degraded state of aDNA. To date, a total of eleven different 16S rRNA gene universal primer pairs have been applied in studies of ancient microbiomes. We analyzed ten of these primer pairs (all primer pairs generating amplicons <500 bp at 99% CI), as well as four additional primer pairs widely used today in ecological studies, for these metrics *in silico* using PrimerProspector^48^ and SILVA^25^, a database containing more than 1.5 million 16S rRNA gene sequence entries (Table 3; Supplementary Table 2). Overall, the following results indicate that irrespective of the variable region being targeted, accurate reconstruction of community profiles may be limited dependent on degree of aDNA fragmentation, taxonomic coverage of primers, and degree of length variation of primers.

#### Amplicon taxonomic resolution

Amplicon resolution here is defined as the degree to which amplified sequences allow the discrimination of distinct taxa (OTUs). Using the SILVA SSU 111 database, clustering of all 16S rRNA sequences at 97% similarity threshold yields a total of 138,462 OTUs, representing the maximum taxonomic resolution of this gene. Individual 16S rRNA gene variable regions contain only a portion of the total sequence variation, and thus have lower taxonomic resolution. To determine the anticipated taxonomic coverage of each primer pair, predicted amplicons generated from the SILVA SSU 111 database were clustered *de novo* using uclust^51^ and a 97% similarity threshold. The V4 515F/806R primers yield the highest taxonomic resolution of the primers analyzed in this study (Table 3), with 56,463 predicted OTUs (41% of the total). The V6 926F/1046R primers have the next highest resolution (50,892 OTUs, 37% of total), closely followed by the V3 U341F/534R primers (49,397 OTUs, 36% of total). By comparison, the V1-V2 and V5 primers yield amplicons with only moderate resolution (30–32% and 20–23%, respectively) and V1 taxonomic resolution is very poor (<1%). In fact, one V1 primer pair previously used in ancient microbiome studies^31^,^33^ is not predicted by our analysis to yield any amplicons.

#### Amplicon taxonomic coverage

Amplicon taxonomic coverage is here defined as the proportion of species-level taxonomic entries for each phylum in the SILVA SSU 111 16S rRNA database that are predicted to amplify using a given primer pair. The 16S rRNA gene contains numerous point mutations and insertion-deletion sites. Although most of these sequence variants are concentrated within the hypervariable regions, some are found within the “conserved” regions as well. As a result, it is not possible to design truly universal primers for the 16S rRNA gene^52^. Nevertheless, certain primer pairs are “more universal” than others and can amplify a greater diversity and proportion of microbial phyla than others. Among the primers analyzed in this study, the V3 U341F/534R primers have the highest predicted taxonomic coverage for the 14 most important oral microbial phyla (Table 3). As discussed above, these primers are predicted to amplify >97% of the taxa in these phyla, except for Euryarchaeota (91%) and Chlamydiae (71%). Notably, however, the V3 U341F/534R primers do amplify *Methanobrevibacter oralis* and *Chlamydophila pneumoniae*, the only characterized members of Euryarchaeota and Chlamydiae, respectively, in the oral cavity. Thus, the V3 U341F/534R primers have very high predicted taxonomic coverage for the oral microbiome. The V4 515F/806R, V5 785F/907R, and V6 926F/1046R primer pairs also yield relatively good overall taxonomic coverage but exhibit poor amplification for one or more phyla. In the case of the V4 515F/806R primers, this includes the relatively important phyla Actinobacteria (90%) and Spirochaetes (80%). The V5 785F/907R primers poorly amplify minor oral taxa in the phyla Chloroflexi (8%) and Candidate division SR1 (87%), and the V6 926F/1046R primers show poor coverage for Candidate division TM7 (80%), Candidate division SR1 (1%), and Euryarchaeota (18%; and do not amplify *Methanobrevibacter*). Worst of all are the V1 and V1-V2 primers, which are predicted to yield low taxonomic coverage (<50%) for nearly all oral phyla.

#### Amplicon length

Amplicon length is here defined as the full length of the 16S rRNA amplicon, including primers. Primer inclusion is important because amplification requires a DNA target of a length sufficient to include the entire targeted region of interest and primer binding sites. For modern DNA, this is rarely an issue, but for aDNA, which is typically highly degraded and fragmented, only a small fraction of the total aDNA extract may be of sufficient length to allow amplification. Median lengths of aDNA reported from bone are typically less than 100 bp^42^, and because of this primer pairs targeting regions >200 bp are rarely effective in ancient DNA studies. The 16S rRNA gene variable regions differ in length, and with a total amplicon length up to 295 bp, the V4 region is the longest of the individual variable regions (Table 3). Likewise, the V1 (up to 276 bp) and combined V1-V2 (up to 380 bp) regions are similarly long. For the remaining variable regions, the V3 U341F/534R, V5 515F/806R, and V6 926F/1046R primers yield the shortest amplicons, 150–194 bp, 141–146 bp, and 152–167 bp, respectively. While amplicons of this size fall within the range of those that have been successfully amplified from mitochondrial^53^ and microbiome^30^,^35^,^36^ aDNA in the past, they greatly exceed the median length of typical aDNA fragments.

## Discussion

Amplicon deep sequencing of one or more 16S rRNA gene hypervariable regions is a highly economical method for high-throughput taxonomic characterization of the human microbiome. However, this approach requires high quality and well-preserved DNA in order to prevent differential PCR amplification and consequent biases in downstream taxonomic analyses. As such, the method has limited applicability for genetic investigations of ancient human microbiomes, where the aDNA is known to be highly degraded and fragmented. For example, we find that median DNA fragment lengths within archaeological dental calculus are less than half (41–50%) the required template length for amplifying the commonly targeted 16S rRNA V3 region. Moreover, this problem is further exacerbated when the targeted hypervariable region contains extensive length polymorphisms, as is true for the V1, V2, and V3 regions. In such cases, the effects of differential PCR amplification are not random, but rather are biased toward taxa with the shortest amplicon lengths. In the case of the universal V3 U341F/534R primers, archaeological specimens tend to produce taxonomic profiles with unusually high frequencies of *Methanobrevibacter*, and to a lesser extent Anaerolinaceae and TM7, all taxa with very short V3 sequences. At the same time, these taxonomic profiles typically have unusually low frequencies of taxa with very long V3 sequences, such as *Treponema*, *Neisseria*, and *Prevotella*. Because *Methanobrevibacter* is the oral taxon with the shortest V3 region (17 bp shorter than the shortest bacterial sequence), it may reach extreme frequencies in some ancient microbiome datasets, even exceeding 60% of the total sequences (e.g., Fig. 1c), a taphonomic artifact we term the “Archaea effect”. Such high frequencies of *Methanobrevibacter* are not observed in corresponding shotgun metagenome datasets (Supplementary Table 3), further confirming that it is an artifact specific to targeted PCR amplification. We have observed that the Archaea effect is characteristic of ancient microbiome taxonomic profiles produced using V3 U341F/534R universal primers from samples with a high degree of endogenous DNA fragmentation, but low exogenous contamination. Other V3 primers, such as 338F/531R, 338F/533R, and 351F/507R do not typically generate this effect because they are less universal and contain primer sequence mismatches that strongly disfavor amplification of Archaea. Nevertheless, the remaining length-based amplification biases documented in other taxa remain applicable.

Although the 16S rRNA gene V3 region is the focus of this study, other hypervariable regions are subject to similar challenges. The V1 and V2 regions exhibit even greater length polymorphisms than the V3 region, and the V4 region, although relatively length invariant, is simply too long (>290 bp) for efficient amplification of aDNA. The V5 785F/907R primer pair performs well on a number of metrics: it has very good predicted taxonomic coverage and is relatively short (144–148 bp) with little amplicon length variation; however, it also contains relatively low information and resulting amplicons have only 57% and 64% of the OTU resolution as the V4 515F/806R and V3 U341F/534R primers, respectively. Perhaps the most promising alternative to the V3 U341F/534R primers is the V6 926F/1046R primer pair; however, it too has drawbacks. The V6 926F/1046R target is relatively short (152–167 bp) with only moderate sequence length variation, and it is predicted to produce amplicons with high taxonomic resolution. However, it has low taxonomic coverage for the bacterial candidate phyla TM7 and SR1, and it is not predicted to amplify the archaeal genus *Methanobrevibacter*. Hypervariable regions V7, V8, and V9, which are not analyzed in this study, are rarely targeted for metataxonomic analysis because of their low information content and poorly conserved primer-binding sites. There are no truly universal 16S rRNA primers, and especially with respect to ancient DNA studies, all metataxonomic approaches involving targeted PCR amplification are likely to be inefficient and consequently at high risk for taxonomic bias as a function of aDNA taphonomy. As such, this greatly limits the utility of this approach in geographic and temporal comparative studies^30^, and even in studies focusing on a single place and time, as observed for the Middenbeemster samples in this study.

The results of this study also have implications for other amplicon-based approaches, such as direct PCR or qPCR of species-specific targets. Species-specific targeted PCR assays have been developed for a number of oral taxa, especially pathogens, and have been used to detect the presence/absence and relative abundance of these bacteria in ancient dental calculus from different temporal periods spanning the Mesolithic to medieval periods^30^,^54^. However, in each case, detection of these taxa depends on the successful amplification of relatively long PCR targets (114 to 179 bp), and thus it is intuitive that taxonomic dropout will increase in samples of higher thermal age simply because they are likely to be more fragmented. For example, the recent reported failure to detect *Streptococcus mutans* in Neolithic and Mesolithic samples compared to Bronze Age samples^30^ cannot be attributed to a true biological absence of this species without first accounting for the possibility that the aDNA in these samples is more highly fragmented, resulting in PCR-dropout. Moreover, attempting to control for this factor through co-amplification of other taxa is problematic when the comparative taxon is (1) present at a higher relative frequency, and/or (2) when primer specificity for the comparative taxon is low, and thus non-specific amplification is possible.

Additional evidence that PCR dropout may skew temporal datasets is evident in a recent study of South American dental calculus samples^54^. The age of the samples ranged from the recent past (ca. 1960–1970) to more than 4,000 years ago, and among the taxa targeted was *Streptococcus gordonii*. This taxon is a highly prevalent and abundant member of the dental plaque microbiome. In the Human Microbiome Project healthy cohort^55^, it is present in 100% of the 104 dental plaque samples, and it is characterized as a common inhabitant of the oral cavity with an observed relative abundance of 1.4% in the HOMD^40^. Among the ancient South American dental calculus samples, more than half of the samples (22/38) yielded no amplification for any of the bacterial targets, and of those that did amplify at least one target, *S. gordonii* was observed in only 75% of the samples. As discussed by the authors of the study, DNA degradation has likely limited PCR amplification in this sample set. Thus, while the detection of an ancient microbe within a sample can be taken as evidence of its presence, the failure to detect an ancient microbe cannot be used to confirm its absence.

While the disadvantages of 16S rRNA V3 length polymorphisms in ancient microbial amplicon metataxonomics are clear, there are also potential advantages. First, because modern microbial contaminants are expected to preferentially amplify over highly degraded and fragmented aDNA, analysis of V3 amplicons should provide a conservative method for qualitatively estimating the relative proportion of endogenous DNA sequences within an ancient microbial sample. Second, amplification bias towards ancient taxa with shorter V3 regions provides information about the approximate size of the amplifiable ancient DNA molecules in the sample. This information can be useful for planning downstream shotgun metagenome analyses, including both ancient sample selection and selection of appropriate sequencing chemistry.

It is clear that quantitative characterization of oral microbiome taxa using either universal or species-specific targeted PCR is problematic for archaeological microbiome samples. Stochastic taxonomic skew resulting from differential PCR amplification is expected for degraded and highly fragmented aDNA in general, and non-random bias toward shorter amplicons is expected for PCR targets containing length polymorphisms. With respect to the 16S rRNA gene, we conclude that extensive length polymorphisms in the V3 region are an important cause of amplification dropout and taxonomic bias in ancient microbiome reconstructions based on this hypervariable region. When using the universal V3 U341F/534R primers, such reconstructions may contain archaeal frequencies in excess of 60%, a clearly non-biological pattern attributable to taphonomy. Such systematic amplification bias confounds attempts to accurately reconstruct microbiome taxonomic profiles from 16S rRNA gene amplicon data. Thus, amplicon metataxonomics, the most commonly used method for comparative microbiome characterization in living humans and primates, as well as microbial ecology studies in general, cannot be applied to ancient samples in a simple and straightforward manner.

Given the highly degraded and fragmented nature of aDNA and the associated difficulties of amplicon metataxonomics, shotgun metagenomics presents a viable alternative for ancient microbiome characterization. Shotgun metagenome sequencing is a powerful molecular approach made possible by massively parallel NGS that allows complex microbiomes to be comprehensively sampled and characterized for microbial structure and diversity. Unlike amplicon-based approaches, shotgun metagenomics is not compromised by short DNA fragment lengths, and in fact it operates with high efficiency on DNA templates of the size range typical of aDNA. Additionally, because it is a non-targeted approach, it is not affected by loci-specific length polymorphisms. Thus, performing shotgun-based metataxonomic analysis by filtering and analyzing 16S rRNA gene sequences from metagenomic datasets presents a tractable compromise – it avoids fragmentation-driven amplification biases while taking advantage of the extraordinary comparative databases available for 16S rRNA gene sequences.

The results generated using this approach can then be complemented by parallel analyses relying on databases of full or partial genome sequences, such as Kraken^56^, MetaPhlAn^57^, MEGAN^58^, and MG-RAST^59^. While these latter approaches utilize information across the entire genome and enable functional potential analysis^60^, they have drawbacks with respect to metataxonomics, in part due to the much smaller scope of genomic databases compared to 16S rRNA gene databases. For example, while 16S rRNA gene sequences in curated databases currently number in the millions (e.g., Greengenes^26^: 1,049,116; SILVA SSU ref. 25: 1,756,783; RDP^61^: 3,224,600), the number of completely or partially (scaffold-stage) sequenced microbial genomes in Genbank is orders of magnitude fewer, numbering less than twenty thousand (search performed September 2015). As a result, taxonomic analysis of metagenomes based on genomic databases is likely to underassign sequences from taxa that are underrepresented or absent in these databases, especially from phyla with few or no cultured members, such as TM7 and Synergistetes. Additionally, this analysis is likely to misassign sequences from common soil microbes, such as *Mycobacterium* spp. and *Yersinia* spp., to pathogenic relatives (e.g., *M. tuberculosis* and *Y. pestis*) that have higher representation in genomic databases. Further challenges of metagenomic taxonomic analysis have been reviewed in detail elsewhere^60^. As genomic databases improve, such problems will lessen, but in the meantime 16S rRNA sequences remain highly valuable in metataxonomic analysis. In the case of ancient DNA, our study suggests that such 16S rRNA gene sequences are best generated by a metagenomic approach, rather than a targeted amplicon approach.

Finally, it is important to note that while microbial community reconstruction from shotgun metagenome data is robust to many of the taphonomic challenges posed by ancient DNA, it is susceptible to other known biases, including variations in GC content^62^,^63^ and genome length^64^,^65^. However, these biases are not specific to ancient DNA, but rather are inherent to current library construction and NGS sequencing methods. Thus, while shotgun metagenome sequencing provides a promising alternative to amplicon sequencing for ancient microbiome samples, one must continue to evaluate these sources of bias when interpreting metagenomic datasets.

In conclusion, while amplicon-based approaches may be useful for qualitatively assessing sample preservation and contamination, they are vulnerable to taphonomic artifacts and are not appropriate for the reconstruction of ancient microbial communities. Instead, we recommend generating community taxonomic data using shotgun metagenome sequencing for ancient microbiome characterization.

## Methods

### Samples

Dental calculus for targeted sequencing was obtained from human skeletal remains (n = 107) originating from: Middenbeemster, the Netherlands (n = 76); Rupert’s Valley, St. Helena (n = 15); Anse à la Gourde, Guadeloupe (n = 5); Lavoutte, St. Lucia (n = 5); Tickhill, Yorkshire, UK (n = 4); Samdzong, Nepal (n = 1); and Camino del Molino, Spain (n = 1). A subset of these samples (n = 4) was also selected for non-targeted sequencing (Table 1). Dental calculus was sampled according to previously described methods^36^.

### DNA extraction

All samples except those from Tickhill were extracted in a dedicated ancient DNA laboratory at the Laboratories of Molecular Anthropology and Microbiome Research (LMAMR) in Norman, Oklahoma, U.S.A. The Tickhill samples were extracted in a dedicated ancient DNA laboratory at the University of York. Both labs operate in accordance with established contamination control precautions and workflows. Non-template extraction controls and reagent blanks were processed in parallel to screen for modern contamination during laboratory procedures. The positive pressure Class 7 ancient DNA clean rooms are physically separated from all laboratories in which PCR is performed. Full body Tyvek suits, masks and gloves were worn to prevent contamination. Buffers and reagents were decontaminated using published protocols^66^. For all samples except Tickhill, DNA extraction was performed as described by Warinner and colleagues^36^ (Extraction Method A, preceded by EDTA decontamination). In brief, 10–20 mg of dental calculus were agitated in 1 ml 0.5M EDTA for 15 minutes to remove surface contaminants. The decontaminated dental calculus was then decalcified in a solution of 0.45M EDTA and 10% proteinase K (Qiagen, the Netherlands) at 55 °C for 8–12 hours and then at room temperature for 5 days. Following centrifugation, the supernatant was extracted for DNA by phenol:chloroform:isoamyl alcohol (25:24:1) extraction. The extracted DNA was isolated by silica purification^67^ and quantified using a Qubit fluorometer. The Tickhill samples were extracted according to the protocol of Rohland and colleagues^68^ and also quantified using a Qubit fluorometer (Supplementary Table 1).

### 16S rRNA amplicon Illumina library preparation and sequencing

Approximately 5 ng of ancient DNA was used to build each targeted library at the LMAMR and at York. Samples, negative extraction controls, and reagent blanks were PCR-amplified using primer constructs containing the universal 16S rRNA V3 region U341F/534R primers and Illumina adapter sequences. Golay barcodes were also included in each reverse primer to allow for sample pooling^28^. Each PCR reaction contained 9.25 μl molecular grade water, 5 μl 5x Phusion buffer, 1 μL 2.5 mg/ml BSA, 2.5 μL 10 mM decontaminated dNTPs, 0.5 μl 10 μM primer 341F, 1.0 μl 10 μM primer 534 R, 0.25 μl Phusion Hot Start II DNA polymerase (2 U/μl) and 1.0 μl of DNA template (5 ng/μl) for a total volume of 20 μl. The temperature profile for the reactions included an initial activation of the enzyme at 98 °C for 30 seconds, followed by 35 cycles of 98 °C for 15 seconds, 52 °C for 20 seconds, 72 °C for 20 seconds, followed by a final 5-minute extension at 72 °C. PCR products were then visualized on 2% agarose gel. For each sample, the PCR products of three successful amplifications were pooled. The pools were then combined and purified using a Qiagen MinElute column. The resulting pooled DNA was quantified using a NanoDrop spectrophotometer, and size selection of the amplicons was performed using a PippinPrep. Prior to sequencing, the amplicon size distribution and successful removal of dimer peaks was confirmed using a Bioanalyzer High Sensitivity DNA assay. All libraries except for those from Tickhill were sequenced using Illumina MiSeq v2 2 × 150 bp chemistry at the Yale Center for Genome Analysis. The Tickhill samples sequenced using Illumina2 × 250 bp chemistry at the Wellcome Trust Sanger Institute. Sequencing depths are provided in Supplementary Table 4.

### Shotgun metagenomic Illumina library preparation and sequencing

Approximately 100 ng of ancient DNA was built into each Illumina shotgun library at the Center for GeoGenetics, Copenhagen, Denmark using the NEBNext DNA Library Prep Master Set (E6070) and blunt-end modified Illumina adapters^69^. The protocol followed the manufacturer’s instructions with minor modifications. Nebulization was skipped. End-repair was performed in 50 μl reactions with 30 μl of DNA extract. The end-repair cocktail was incubated for 20 min at 12 °C and 15 min at 37 °C and purified using Qiagen MinElute silica spin columns following the manufacturer’s instructions and eluted in 30 μl. After end-repair, Illumina-specific adapters^69^ were ligated to end-repaired DNA in 50 μl reactions. The reaction was incubated for 15 min at 20 °C and purified using Qiagen QiaQuick columns before elution in 30 μl EB. The adapter fill-in reaction was performed in a final volume of 50 μl and incubated for 20 min at 37 °C followed by 20 min at 80 °C to inactivate the Bst polymerase. Libraries were amplified and indexed in a 50 μl PCR reaction, using 15 μl of library template, 25 μl of a 2x KAPA U+ master mix, 5.5 μl H2O, 1.5 μl DMSO, 1 μl BSA (20 mg/ml), and 1 μl each of a forward and reverse indexing primer (10 μM). Thermocycling conditions were 5 min at 98 °C, followed by 10–12 cycles of 15 sec at 98 °C, 20 sec at 60 °C, and 20 sec at 72 °C, and a final 1 min elongation step at 72 °C. Amplified libraries were purified using Agencourt AMPure XP beads, and eluted in 30 μl EB. The size distribution of the full Illumina-compatible construct was estimated using an Agilent Bioanalyzer. Libraries were pooled in equimolar amounts sequenced using v2 2 × 100 bp chemistry on a single lane of the Illumina HiSeq 2000. Sequencing depths are provided in Supplementary Table 4. 16S rRNA reads represent 0.2% of total shotgun sequencing reads.

### 16S rRNA gene amplicon data analysis

Read pairs were quality filtered, trimmed, and merged to reconstruct the full V3 region using ‘PEAR’^70^. Briefly, sequences with ambiguous bases (‘N’) were removed, and reads were quality filtered to remove bases with a Phred score <30. These merged read pairs were demultiplexed in QIIME, followed by closed-reference OTU assignment using ‘uclust’^51^. A sequence similarity threshold of 97% was used to assign reads to OTUs against the Greengenes 13.8 database^26^ as a reference. The resulting OTU tables were not rarefied (in order to retain the low read count control samples), and summarized at the taxonomic levels of phylum and genus. Oral-associated genera were determined based on presence or absence in the HOMD. Bayesian microbial source tracking was performed using SourceTracker^41^, with the inclusion of three classes of soil, gut, skin and oral samples as sources. The published datasets used as sources in this analysis are provided in Supplementary Table 5. Read statistics are summarized in Supplementary Table 4. Data for Figure 1 is available in Supplementary Data 1.

### Shotgun metagenome data analysis

Illumina adapters were removed from paired end reads using ‘Cutadapt’^71^. Trimmed reads were then processed using ‘Sickle’^72^ to trim low quality bases (Phred score <30), remove reads shorter than 25 bp, and remove reads with ambiguous bases. The resulting forward and reverse read pairs were reordered using custom Perl scripts. Read pairs were aligned locally (no soft clipping of ends) against the SILVA SSU 111 reference dataset using Bowtie2^73^ (–no-unal–local). Resulting alignment files were processed using ‘samtools’^74^, followed by Picard-tools to generate fragment-length statistics (default parameters). Additionally, reads mapping to the 16S rRNA gene were recovered from the alignment files, and assigned to OTUs following the closed-reference OTU protocol in QIIME v.1.8 using default settings and Greengenes 13.8 as the reference database, similar to the V3 amplicon dataset. Read statistics are summarized in Supplementary Table 4. In addition to 16S read analysis, genus level taxonomic summaries (Supplementary Table 6) were also generated from phylogenetically informative single copy marker loci as previously described^75^.

Ancient DNA damage profiles were explored by mapping shotgun metagenomic reads to reference genomes for common Gram-positive (*Streptococcus gordonii* and *Propionibacterium propionicum*) and Gram-negative (*Lautropia mirabilis* and *Porphyromonas gingivalis*) oral bacteria found among all the four ancient calculus samples. Read mapping was performed using Bowtie2^76^ (–no-unal –local), followed by removal of PCR and optical duplicates using Picard-tools. The resulting alignment file was used to generate fragment-length statistics using Picard-tools, and to characterize DNA damage patterns using mapDamage (v2.0)^77^.

### *In silico* analysis 16S rRNA gene V3 and V4 regions

16S rRNA secondary structures for *E. coli*, *M. oralis*, *C. diphtheria*, and *S. pyogenes* were retrieved from the Comparative RNA Web Site and Project^78^. To investigate the V3 and V4 regions of the 16S rRNA gene, the SILVA SSU 111 database was queried using PrimerProspector v 1.0.1^48^ and the primers U341F/534R (V3) and 515F/806R (V4), to retrieve the corresponding sequences. Length distribution statistics were generated for the extracted V3 and V4 region sequences using the R statistical package, with limits of length variation determined using 99% confidence intervals (to reduce impact of chimeras and mispriming). Sequence length distribution was also represented as density plots, generated in R. To ensure the multimodal distribution of V3 is not a feature unique to the SILVA SSU 111 database, *in silico* amplicons were also generated for the V3 region using the SILVA SSU 115, Greengenes 13.8, NCBI’s 16S collection (last updated 7/30/2013), and the RDP 11.3 databases (Supplementary Figure 3). Sequence length distribution was represented as density plots as describe above. To further investigate V3 length variation at the genus level, we selected 31 representative oral genera from 9 major microbial phyla from the SILVA SSU 111 dataset and used the R statistical package to plot the length distribution of OTUs assigned to each genus in a heatmap. The genera are organized into a cladogram according to NCBI taxonomy. Log fold changes in observed frequency between V3 amplicon and shotgun metagenome datasets from archaeological dental calculus samples was calculated for these genera.

### Thermal age modeling

Thermal age modeling was performed using the JRA 1: PrediCtoR tool^43^. We used as our starting oral microbiome community HOMD frequency data for a select group of oral taxa provided in Table 2. Thermal ages were determined for the seven archaeological sites included in this study (Table 1) and applied to this model. Model parameters for estimating the probability of a nucleotide being damaged (λ) included archaeological site age, longitude, latitude, altitude, sediment type, and effective burial temperature. We calculated the probability of a DNA fragment of size x or greater being present as e^−λ*x*^. We applied this probability to the frequency of each taxon in our starting community and predicted resulting taxon frequency for the seven thermal age λ values.

### *In silico* analysis of primers

The position of the primer start and stop coordinates are reported relative to *Escherichia coli* (Genbank accession J01695). *In silico* amplicons for each primer pair were generated using PrimerProspector 1.0.1^48^ and the SILVA SSU 111^25^ and Greengenes 13.8^26^ databases as references. The PrimerProspector settings were as follows: 3′ length: 5 bp; non 3′ mismatch penalty: 0.40 per mismatch; 3′ mismatch penalty: 1.00 per mismatch; last base mismatch penalty: 3.00; non 3′ gap penalty: 1.00 per gap; and 3′ gap penalty: 3.00 per gap. To pass, primer alignment to the reference must be ≤1.00. Thus, alignments passed with a single non-3′ gap, a 3′ mismatch that is not the final base, or two non-3′ mismatches. The resulting amplicons were analyzed for length variation, taxonomic resolution and taxonomic coverage. Sequence length variation was analyzed using the R statistical package. Taxonomic resolution was inferred from the number of OTUs generated through *de novo* clustering of the amplicons at 97% sequence similarity using ‘uclust′^51^ as implemented in QIIME v.1.8^39^ was used to analyze the taxonomic resolution. The number of OTUs generated from the amplicons was compared to the total number of OTUs generated from full-length 16S rRNA gene sequences (138,462 OTUs). Taxonomic coverage was retrieved from the results of PrimerProspector^48^ (see above).

## Additional Information

**Accession codes:** Genetic data have been deposited in the NCBI Short Read Archive (SRA) under the project accession PRJNA278036. Sample accession IDs are provided in Supplementary Table 4.

**How to cite this article**: Ziesemer, K. A. *et al.* Intrinsic challenges in ancient microbiome reconstruction using 16S rRNA gene amplification. *Sci. Rep.* **5**, 16498; doi: 10.1038/srep16498 (2015).

## Supplementary Material

Supplementary Information

Supplementary Dataset 1

## Figures and Tables

**Table 1:** Archaeological dental calculus samples analyzed in this study.

Geographic origin	Time Period	ThermalAge λ^a^	Ampliconsequencing (N)^b^	Shotgunsequencing (N)	Shotgunsample ID^c^
Rupert’s Valley, St. Helena	1840–1872 CE^f^	0.0030	15	—	—
Middenbeemster, Netherlands	1611–1866 CE^d^	0.0006	76	1	454C^e^
Tickhill, Yorkshire, UK	ca. 1450–1600 CE^i^	0.0021	4	—	—
Anse à la Gourde, Guadeloupe	ca. 975–1395 CE^g^	0.0701	5	1	F1948C
Lavoutte, St. Lucia	ca. 990–1255 CE^h^	0.0863	5	—	—
Samdzong, Nepal	ca. 400–650 CE^j^	0.0045	1	1	37C^k^
Camino del Molino, Spain	ca. 2340–2920 BCE^l^	0.0149	1	1	214C^m^

^a^Mean thermal age λ calculated using the JRA 1: PrediCtoR tool hosted on www.thermal-age.edu^43^.

^b^See Supplementary Table 1 for full list of analyzed samples.

^c^The suffix “C” was added to the sample name to denote that analysis was performed on calculus.

^d^The majority of burials date to CE 1829–1866. Cemetery dates of death were retrieved from Middenbeemster municipal records^82^. Sample S454C is from an individual with a recorded death date of CE 1856.

^e^Sample ID abbreviated from S454V0963. See Supplementary Table 1.

^f^Cemetery dates are based on historical records^83^.

^g^Burial dating to the Caribbean Late Ceramic Age^84^,^85^. See Supplementary Table 1 for radiocarbon dates.

^h^Burials dating to the Caribbean Late Ceramic Age^86^. See Supplementary Table 1 for radiocarbon dates.

^i^Cemetery dates of death (CE 1412–1532) were retrieved from St. Mary’s Church, Tickhill.

^j^Shaft tomb burial dating to the Samdzong period^87^,^88^,^89^. See Supplementary Table 1 for radiocarbon dates.

^k^Sample name has been abbreviated from 37.UM2010.9. See Supplementary Table 1.

^l^Burial dating to the Chalcolithic period. Time period is inferred from radiocarbon dated associated skeletal material^90^. See Supplementary Table 1 for radiocarbon dates.

^m^Sample ID abbreviated from CMOL214.

**Table 2:** 16S rRNA V3 (U341F/534R) amplicon lengths for oral microbiome taxa of interest.

Oral taxa of interest	16S rRNA V3amplicon length (bp)	Rank abundance inHOMD
Oral commenals		
*Actinomyces naeslundii*	190	0.23%
*Anaerolineae G-1* sp.^a^	169	<0.01%
*Atopobium parvulum*	173	0.30%
*Campylobacter gracilis*	169	1.04%
*Corynebacterium matruchotii*	189	0.49%
*Eubacterium saphenum*	171	0.37%
*Fusobacterium nucleatum*	172	1.35%
*Methanobrevibacter oralis*	151	*
*Mogibacterium timidum*	171	0.27%
*Neisseria mucosa*	193	0.33%
*Oribacterium sinus*	168	0.14%
*Parvimonas micra*	168	0.68%
*Prevotella melaninogenica*	188	0.22%
*Rothia dentocariosa*	173	0.60%
*Streptococcus mitis*	194	5.66%
TM7 spp.^b^	168	0.24%
*Veillonella parvula*	194	6.63%
Caries pathogens		
*Lactobacillus salivarius*	193	0.09%
*Streptococcus mutans*	194	4.31%
Periodontal pathogens		
*Aggregatibacter actinomycetemcomitans*	193	0.03%
*Filifactor alocis*	171	0.31%
*Porphyromonas gingivalis*	188	0.26%
*Tannerella forsythia*	188	0.25%
*Treponema denticola*	194	0.22%
Respiratory pathogens		
*Bordetella pertussis*	193	**
*Corynebacterium diphtheriae*	173	**
*Haemophilus influenzae*	193	0.29%
*Streptococcus pneumoniae*	194	0.18%
*Streptococcus pyogenes*	194	**
Systemic pathogens		
*Neisseria meningitidis*	193	0.17%
*Mycobacterium tuberculosis*	185	**
*Mycobacterium leprae*	185	**
*Treponema pallidum*	194	**
Dietary		
*Streptophyta (plant chloroplast)*	170	***

*Rank abundance could not be determined for this taxon because the HOMD primers do not amplify archaeal sequences^50^.

**Taxon below detection limits of molecular cloning in HOMD.

***Chlorplast DNA not included in HOMD rank abundance calculation.

^a^Within Chlorflexi, the bacterial class Anaerolineae contains several oral taxa that have not yet been formally described, and naming of these taxa is inconsistent across databases. We observed one of these Anaerolineae taxa in both our modern and ancient datasets, and its 16S rRNA sequence is identical to *Anaerolineae G-1* (HOMD oral taxon ID 439) and *SHD-231* (Greengenes 13).

^b^TM7 is a bacterial candidate phylum without cultured members.

**Table 3:**
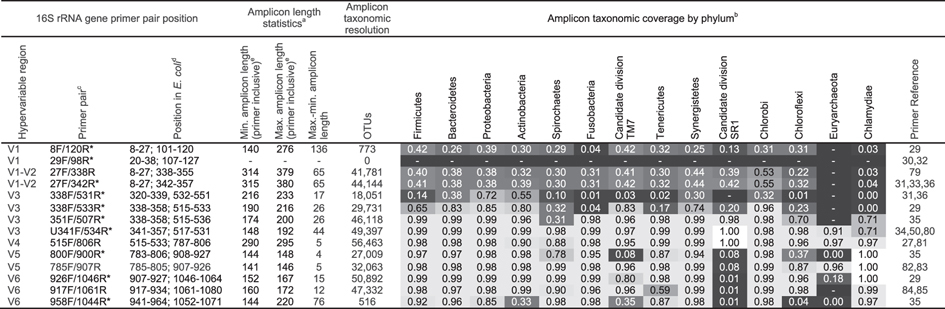
16S rRNA gene primer pair information and *in silico* amplicon statistics.

*Primer pair has been used in previous ancient microbiome studies. ^a^Length statistics obtained from SILVA SSU 111 database. ^b^*In silico* estimate using PrimerProspector^48^ of the proportion of sequences in SILVA SSU 111 database (http://www.arb-silva.de) that the primer pair will amplify per phylum. Phyla are sequentially ordered from left to right by the number of species entries per phylum in the HOMD^40^. ^c^Primer pair naming conventions have changed through time and are not consistent across studies. ^d^Position of primer start and stop coordinates relative to *Escherichia coli* Genbank accession J01695. Full primer sequences are provided in Supplementary Table 2. ^e^Reported for 99% CI of the SILVA SSU 111 database. Outliers, which may include chimeras, are excluded. ^f^OTUs generated per primer pair by *de novo* clustering of the Silva 111.1 database at 97% similarity. The total number of OTUs generated from full 16S rRNA gene sequences is 138,462.
