# Corrigendum: Intrinsic challenges in ancient microbiome reconstruction using 16S rRNA gene amplification

**DOI:** 10.1038/srep27163

**Published:** 2016-06-02

**Authors:** Kirsten A. Ziesemer, Allison E. Mann, Krithivasan Sankaranarayanan, Hannes Schroeder, Andrew T. Ozga, Bernd W. Brandt, Egija Zaura, Andrea Waters-Rist, Menno Hoogland, Domingo C. Salazar-García, Mark Aldenderfer, Camilla Speller, Jessica Hendy, Darlene A. Weston, Sandy J. MacDonald, Gavin H. Thomas, Matthew J. Collins, Cecil M. Lewis, Corinne Hofman, Christina Warinner

Scientific Reports 5: Article number: 16498;10.1038/srep16498 Published online: 11 13 2015; Updated: 06 02 2016

The Acknowledgements section in this Article is incomplete.

“This work was supported by the European Research Council (FP7 ERC-Synergy Nexus1492 project grant number 319209) and the US National Institutes of Health (R01 GM089886), the BBVA Foundation (I Ayudas a Investigadores, Innovadores y Creadores), the Generalitat Valenciana (VALi+d APOSTD/2014/123 and GV/2015/060), the European Union (EUROTAST FP7 PEOPLE-2010 MC ITN, Braudel-IFER-FMSH; FP7/2007-2013 - MSCA-COFUND, n°245743), and the Centre for Chronic Diseases and Disorders (C2D2) Research Priming Fund grant to CFS. C2D2 is part-funded by the Wellcome Trust (ref: 097829). The authors thank Joaquín Lomba for his assistance with obtaining samples; Amanda Henry for providing helpful comments on the manuscript; Jacqueline Eng for providing osteological data for the Samdzong sample; María Haber and Azucena Avilés for providing osteological data for the Camino del Molino sample; Malin Holst for providing osteological data for the Tickhill samples; and also Matthias Garn & Partner, the Parish Church Council for St. Mary’s Church, Tickhill, South Yorkshire, and Wiles & Maguire, Ltd., for assistance with sample provisioning.”

should read:

“This work was supported by the European Research Council (FP7 ERC-Synergy Nexus1492 project grant number 319209), the US National Science Foundation (BCS-1516633) and the US National Institutes of Health (R01 GM089886), the BBVA Foundation (I Ayudas a Investigadores, Innovadores y Creadores), the Generalitat Valenciana (VALi+d APOSTD/2014/123 and GV/2015/060), the European Union (EUROTAST FP7 PEOPLE-2010 MC ITN, Braudel-IFER-FMSH; FP7/2007-2013 - MSCA-COFUND, n°245743), and the Centre for Chronic Diseases and Disorders (C2D2) Research Priming Fund grant to CFS. C2D2 is part-funded by the Wellcome Trust (ref: 097829). The authors thank Joaquín Lomba for his assistance with obtaining samples; Amanda Henry for providing helpful comments on the manuscript; Jacqueline Eng for providing osteological data for the Samdzong sample; María Haber and Azucena Avilés for providing osteological data for the Camino del Molino sample; Malin Holst for providing osteological data for the Tickhill samples; and also Matthias Garn & Partner, the Parish Church Council for St. Mary’s Church, Tickhill, South Yorkshire, and Wiles & Maguire, Ltd., for assistance with sample provisioning.”

