# Supplementary material for: Intrinsic challenges in ancient microbiome reconstruction using 16S rRNA gene amplification
Source: Sci Rep. 2015 Nov 13;5:16498. doi: 10.1038/srep16498 (PMC4643231; doi:10.1038/srep16498)
Supplement: Supplementary Information [file srep16498-s1.pdf]

Supplementary Information:

Intrinsic challenges in ancient microbiome reconstruction using 16S rRNA gene amplification

Kirsten A. Ziesemer[1], Allison E. Mann[2], Krithivasan Sankaranarayanan[2], Hannes Schroeder[1,3], Andrew T. Ozga[2], Bernd W. Brandt[4], Egija Zaura[4], Andrea Waters-Rist[1], Menno Hoogland[1], Domingo C. Salazar-García[5-7], Mark Aldenderfer[8], Camilla Speller[9], Jessica Hendy[9], Darlene A. Weston[1,10], Sandy J. MacDonald[11], Gavin H. Thomas[11], Matthew J. Collins[9], Cecil M. Lewis, Jr.[2], Corinne Hofman[1], Christina Warinner[2]*

[1]Faculty of Archaeology, Leiden University, Einsteinweg 2, 2333 CC, Leiden, the Netherlands
[2]Department of Anthropology, University of Oklahoma, Norman, OK, USA
[3]Center for Geogenetics, University of Copenhagen, Denmark
[4]Department of Preventive Dentistry, Academic Center for Dentistry Amsterdam, University of Amsterdam and VU University Amsterdam, the Netherlands
[5]Department of Anthropology, University of Cape Town, South Africa
[6]Departament de Prehistòria i Arqueologia, Universitat de València, Spain
[7] Department of Human Evolution, Max-Planck Institute for Evolutionary Anthropology, Leipzig, Germany
[8]School of Social Sciences, Humanities, and Arts, University of California, Merced, USA
[9]Department of Archaeology, University of York, York, UK
[10]Department of Anthropology, University of British Columbia, Vancouver, Canada
[11]Department of Biology, University of York, York, UK

*Corresponding author

Correspondence to: twarinner@gmail.com

Supplementary Table 1 | Archaeological dental calculus specimens analyzed by amplicon sequencing

Site	Sample ID[a]	Age[b]	Sex[c]	Calculus score[d]	DNA yield (ng/mg)
Middenbeemster	S029V0024	-	-	3	47.0
Middenbeemster	S047V0045	EYA	F	2	23.4
Middenbeemster	S051V0059	OA	M	2	39.7
Middenbeemster	S054V0051	-	-	2	59.4
Middenbeemster	S059V0133	MA	M	2	54.0
Middenbeemster	S060V0037	EYA	F	3	70.2
Middenbeemster	S100V0159	OA	M	3	44.3
Middenbeemster	S101V0131	LYA	F	3	108.6
Middenbeemster	S121V0211	MA	PF	3	16.6
Middenbeemster	S151V0666	LYA	F	1	72.8
Middenbeemster	S153V0435	MA	M	3	82.0
Middenbeemster	S155V0196	OA	M	3	54.3
Middenbeemster	S160V0613	LYA	F	1-2	34.6
Middenbeemster	S174V0408	MA	PF	3	54.8
Middenbeemster	S183V0311	LYA	F	1	10.8
Middenbeemster	S194V0440	OA	M	3	81.6
Middenbeemster	S216V0233	MA	F	2	77.1
Middenbeemster	S226V0282	LYA	M	2	66.5
Middenbeemster	S233V0304	OA	M	3	68.1
Middenbeemster	S236V0335	EYA	M	2	81.8
Middenbeemster	S239V0369	EYA	M	2-3	42.5
Middenbeemster	S240V0362[e]	MA	M	2	57.6
Middenbeemster	S242V0338	OA	M	2	46.3
Middenbeemster	S243V0381	OA	F	3	57.9
Middenbeemster	S249V0394	LYA	M	2-3	54.1
Middenbeemster	S251V0624	MA	M	1	45.7
Middenbeemster	S263V0445	MA	M	3	37.1
Middenbeemster	S270V1067	EYA	M	2	113.7
Middenbeemster	S275V0526[f]	-	-	2	36.7
Middenbeemster	S281V0542[e]	MA	PM	3	29.8
Middenbeemster	S285V0452	OA	M	3	103.1
Middenbeemster	S290V0472	EYA	M	3	51.8
Middenbeemster	S306V0561	LYA	M	2	<0.5
Middenbeemster	S307V0591	EYA	F	3	35.5
Middenbeemster	S311V0956[e]	EYA	F	3	<0.5
Middenbeemster	S313V0926	MA	M	2	22.4
Middenbeemster	S325V0670	LYA	M	2	57.2
Middenbeemster	S342V0737	OA	M	2	23.2
Middenbeemster	S345V0757	LYA	F	1	<0.5
Middenbeemster	S347V0741	OA	M	3	44.4
Middenbeemster	S350V0884	EYA	F	2-3	28.8
Middenbeemster	S355V0748	OA	PF	3	23.3
Middenbeemster	S356V0864	OA	F	3	34.7
Middenbeemster	S363V0766	OA	M	2	63.0
Middenbeemster	S371V0790[f]	MA	M	2	80.6
Middenbeemster	S372V0808	-	F	2-3	67.1
Middenbeemster	S379V0851	EYA	M	2	101.3
Middenbeemster	S380V0821	MA	M	3	83.6
Middenbeemster	S383V0880	MA	F	1	129.0
Middenbeemster	S399V0872	MA	PM	3	39.7
Middenbeemster	S402V0907	MA	M	3	107.1
Middenbeemster	S405V0882	MA	F	3	57.4
Middenbeemster	S420V0936	LYA	F	3	44.8
Middenbeemster	S422V0962	LYA	F	3	71.2
Middenbeemster	S427V0938	LYA	M	2-3	32.9
Middenbeemster	S430V0965	LYA	F	2	22.7
Middenbeemster	S432V0981	LYA	M	2	69.8
Middenbeemster	S441V0932	EYA	F	2	39.4
Middenbeemster	S454V0963[g]	EYA	M	2	60.5
Middenbeemster	S460V0971	-	-	2	100.7

Site	Sample ID[a]	Age[b]	Sex[c]	Calculus score[d]	DNA yield (ng/mg)
Middenbeemster	S464V1012[e]	MA	M	2	42.0
Middenbeemster	S466V1010	MA	F	2	48.4
Middenbeemster	S467V1022	LYA	M	3	20.7
Middenbeemster	S473V1003	LYA	M	2	46.8
Middenbeemster	S474V0998	OA	PF	3	59.0
Middenbeemster	S482V1048	MA	M	3	65.5
Middenbeemster	S497V1059	LYA	M	2	<0.5
Middenbeemster	S498V1071	OA	F	2	23.9
Middenbeemster	S501V1097	EYA	F	2-3	28.8
Middenbeemster	S502V1062	EYA	M	2	118.7
Middenbeemster	S504V1109	MA	F	3	21.9
Middenbeemster	S511V1126[f]	OA	M	2	11.5
Middenbeemster	S512V1105[f]	MA	F	1	22.7
Middenbeemster	S518V1086[f]	OA	PF	3	14.3
Middenbeemster	S520V1118	OA	M	3	37.3
Middenbeemster	S524V1120	MA	M	3	35.0
Middenbeemster	S538V1164	LYA	PF	1	16.5
St. Helena	STH219	LYA	M	2	1.7
St. Helena	STH268	A	-	2-3	0.5
St. Helena	STH276	EYA	M	2-3	0.9
St. Helena	STH319	A	-	3-4	<0.5
St. Helena	STH423	MA	M	2-3	3.1
St. Helena	STH430	LYA	M	4	0.7
St. Helena	STH419	EYA	F	3-4	2.5
St. Helena	STH284	EYA	F	1-2	3.2
St. Helena	STH358	LYA	M	1-2	2.4
St. Helena	STH218	LYA	PM	2-3	0.6
St. Helena	STH359	EYA	M	1-2	1.4
St. Helena	STH389	EYA	M	1-2	1.9
St. Helena	STH474	MA	M	2	2.1
St. Helena	STH449	LYA	M	2-3	1.7
St. Helena	STH343	MA	M	1-2	1.8
Lavoutte	F67-31[h]	36-45	M	3	3.3
Lavoutte	F67-33	46+	M	3	<0.5
Lavoutte	F68-04[i]	26-35	M	3	13.9
Lavoutte	F69-01	36-45	M	3	1.7
Lavoutte	F69-05[j]	36-45	U	3	5.4
Anse à la Gourde	F0335	Adult	F	3	<0.5
Anse à la Gourde	F0349[k]	26-35	M	3	2.5
Anse à la Gourde	F0430[l]	26-35	F	3	12.5
Anse à la Gourde	F0529	18-25	F	3	<0.5
Anse à la Gourde	F1948[g]	46+	M	1-2	55.3
Tickhill	TKAC	46+	F	1-2	140.7
Tickhill	TKDC	OA	F	3	33.5
Tickhill	TKEC	Adult	M	2	197.3
Tickhill	TKFC	Adult	PM	3	33.5
Samdzong	37.UM2010.9[g,m]	OA	M	3	46.2
Camino del Molino	CMOL214[g,n]	Adult	PF	4	24.7

Notes:
[a]Sample ID refers to the site-specific burial or individual code.
[b]Age determined from analysis by A. Waters-Rist (Middenbeemster), D. Weston (Anse à la Gourde, Lavoutte), J. Eng (Samdzong), M. Haber and A. Avilés (Camino del Molino), A. Witkin and D. Swales (St. Helena), and M. Holst (Tickhill). Age estimated using morphological features, patterns of wear and degree of ossification of the pubic symphysis, auricular surface, cranial sutures, sternal rib end and dentition [1-9]. Age codes: EYA, early young adult; LYA, late young adult; MA, middle-aged adult; OA, older adult; -, not analyzed.
[c]Sex determined from analysis by A. Waters-Rist (Middenbeemster), D. Weston (Anse à la Gourde, Lavoutte), J. Eng (Samdzong), M. Haber and A. Avilés (Camino del Molino), A. Witkin and D. Swales (St. Helena), and M. Holst (Tickhill). Sex estimated using morphological features of the cranium, mandible and pelvic bone [10-15]. Sex codes: F, female; PF, probable female; M, male; PM, probable male; U, undetermined; -, not analyzed.

[d]Calculus severity determined by K. Ziesemer and reported using the Dobney and Brothwell scoring method [2,16].
[e]Unlike the majority of Middenbeemster burials which date to 1829-1866 CE, this individual dates to an earlier period of the cemetery (1617-1829 CE).
[f]Date of individual not determined.
[g]Selected for shotgun sequencing
[h]Associated wood in grave radiocarbon dated to 1000 ± 40 BP (cal CE 975-1155). Calibration program: Calib Rev 7.0.2. Calibration data set: Intcal 13.14c.
[i]Bone collagen radiocarbon dated to 790 ± 35 BP (cal CE 1270-1395). Calibration program: Calib Rev 7.0.2. Calibration data set: Mixed Marine/No. Hem. (30% marine diet).
[j]Bone collagen radiocarbon dated to 960 ± 35 BP (cal CE 1055-1085 [10%] or 1120-1266 [90%]). Calibration program: Calib Rev 7.0.2. Calibration data set: Mixed Marine/No. Hem. (30% marine diet).
[k]Bone collagen radiocarbon dated to 975 ± 30 BP (cal CE 1055-1255). Calibration program: Calib Rev 7.0.2. Calibration data set: Mixed Marine/No. Hem. (30% marine diet).
[l]Bone collagen radiocarbon dated to 1100 ± 35 BP (cal CE 990-1150). Calibration program: Calib Rev 7.0.2. Calibration data set: Mixed Marine/No. Hem. (30% marine diet).
[m]Additional bone collagen from the same tomb (Tomb 1) is radiocarbon dated to 1440 ± 15 BP [17,18].
[n]Additional bone collagen samples from the same burial assemblage are radiocarbon dated to 4170 ± 40 BP, 3850 ± 40 BP, and 3900 ± 40 BP [19].

Supplementary Table 2. 16S rRNA gene primer sequences analyzed in this study

Hypervariable region	Primer pair[a]	Position in *E. coli*[b]	Primer sequences	Reference
V1	8F/120R*[c]	8-27; 101-120	F 5'-AGAGTTTGATYMTGGCTCAG -3'<br>R 5'-TTACTCACCCGTNCGCCRCT-3'	20
V1	29F/98R*	20-38; 107-127	F 5'-TGGCTCAGATTGAACGCTG-3'<br>R 5'-CCAGACATTACTCACCCGTCC-3'	21,22
V1-V2	27F/338R	8-27; 338-355	F 5'-AGAGTTTGATCCTGGCTCAG-3'<br>R 5'-GCTGCCTCCCGTAGGAGT-3'	23
V1-V2	27F/342R*	8-27; 342-357	F 5'-AGAGTTTGATCCTGGCTCAG-3'<br>R 5'-CTGCTGCCTCCCGTAG-3'	24-26
V3	338F/531R*	320-339, 532-551	F 5'-AACTGAGACACGGTCCAGAC-3'<br>R 5'-ACGCTTGCACCCTCCGTATT-3'	25,26
V3	338F/533R*	338-358; 515-533	F 5'-ACTCCTACGGGAGGCAGCAGT-3'<br>R 5'-TTACCGCGGCTGCTGGCAC-3'	20
V3	351F/507R*	338-358; 515-536	F 5'-ACTCCTACGGGAGGCAGCAGT-3'<br>R 5'-GTATTACCGCGGCTGCTGGCAC-3'	27
V3	U341F/534R*	341-357; 517-531	F 5'-CCTACGGGRSGCAGCAG-3'<br>R 5'-ACCGCGGCKGCTGGC-3'	28-30
V4	515F/806R	515-533; 787-806	F 5'-GTGCCAGCMGCCGCGGTAA-3'<br>R 5'-GGACTACHVGGGTWTCTAAT-3'	31,32
V5	800F/900R*	783-806; 908-927	F 5'-CAGGATTAGATACCCTGGTAGTCC-3'<br>R 5'-CCCGTCAATTCCTTTGAGTT-3'	27
V5	785F/907R	785-805; 907-926	F 5'-GGATTAGATACCCBRGTAGTC-3'<br>R 5'-CCGTCAATTCMTTTRAGTTT-3'	33,34
V6	926F/1046R*	907-927; 1046-1064	F 5'-AAACTYAAAKGAATTGACGGG-3'<br>R 5'-CGACARCCATGCASCACCT-3'	20
V6	917F/1061R	917-934; 1061-1080	F 5'-GAATTGACGGGGRCCCGC-3'<br>R 5'-TCACGRCACGAGCTGACGAC-3'	35,36
V6	958F/1044R*	941-964; 1052-1071	F 5'-GGTGGAGCATGTGGTTTAATTCGA-3'<br>R 5'-GAGCTGACGACAGCCATGCA-3'	27

Notes
*Primer pair has been previously used in ancient microbiome studies.
[a]Primer pair naming conventions have changed through time and are not consistent across studies.
[b]Position of primer start and stop coordinates relative to Escherichia coli Genbank accession J01695.
[c]The degenerate base I in the primer sequence was replaced with N prior to PrimerProspector analysis.

Supplementary Table 3. Frequency of *Methanobrevibacter* in archaeological dental calculus analyzed using amplicon (V3 U341F/534R) and shotgun metagenomics approaches

Sample ID	Site	Amplicon	Shotgun
454C	Middenbeemster	12.497%	2.053%
F1948C	Anse à la Gourde	26.131%	2.158%
37C	Samdzong	5.893%	0.627%
214C	Camino del Molino	6.554%	1.643%

Supplementary Table 4 | Amplicon and shotgun sequencing statistics

Sample Name	Library Type	Number of Reads	Number assigned to OTUs	Accession
Modern				
ModCalcControls1	Amplicon	110567	97751	SAMN03400228
ModCalcControls2	Amplicon	106781	94562	SAMN03400229
Middenbeemster				
S047V0045	Amplicon	123589	96150	SAMN03400234
S051V0059	Amplicon	100636	78534	SAMN03400235
S054V0051	Amplicon	100774	77230	SAMN03400236
S059V0133	Amplicon	100815	79359	SAMN03400237
S060V0037	Amplicon	87517	70334	SAMN03400238
S100V0159	Amplicon	68133	48212	SAMN03400239
S101V0131	Amplicon	89568	75559	SAMN03400240
S121V0211	Amplicon	56929	48143	SAMN03400242
S151V0666	Amplicon	74485	65993	SAMN03400243
S153V0435	Amplicon	303279	222300	SAMN03400244
S155V0196	Amplicon	42592	35596	SAMN03400245
S160V0613	Amplicon	105160	88927	SAMN03400246
S174V0408	Amplicon	173469	148124	SAMN03400247
S183V0311	Amplicon	137413	104328	SAMN03400248
S194V0440	Amplicon	58940	49914	SAMN03400249
S216V0233	Amplicon	151125	119385	SAMN03400250
S226V0282	Amplicon	127659	101297	SAMN03400251
S233V0304	Amplicon	105427	86389	SAMN03400252
S236V0335	Amplicon	106176	88127	SAMN03400253
S239V0369	Amplicon	85528	76561	SAMN03400254
S240V0362	Amplicon	151191	134259	SAMN03400255
S242V0338	Amplicon	174853	157259	SAMN03400256
S243V0381	Amplicon	82905	66577	SAMN03400257
S249V0394	Amplicon	160663	144886	SAMN03400258
S251V0624	Amplicon	75591	58050	SAMN03400259
S263V0445	Amplicon	83555	74930	SAMN03400260
S270V1062	Amplicon	70598	61277	SAMN03400261
S275V0526	Amplicon	103173	79146	SAMN03400262
S281V0542	Amplicon	115688	102029	SAMN03400263
S285V0452	Amplicon	98293	73272	SAMN03400264
S290V0472	Amplicon	119224	100402	SAMN03400265
S307V0591	Amplicon	119934	103220	SAMN03400266
S311V0956	Amplicon	82039	71668	SAMN03400267
S313V0926	Amplicon	115055	88275	SAMN03400268
S325V0670	Amplicon	132336	109210	SAMN03400269
S342V0737	Amplicon	51154	41759	SAMN03400270
S345V0757	Amplicon	109945	96770	SAMN03400271
S347V0741	Amplicon	71218	63178	SAMN03400272
S350V0884	Amplicon	98044	82518	SAMN03400273
S355V0748	Amplicon	168412	146504	SAMN03400274
S356V0864	Amplicon	144903	113533	SAMN03400275
S363V0766	Amplicon	150656	128858	SAMN03400276
S371V0790	Amplicon	125794	100469	SAMN03400277
S372V0808	Amplicon	131696	116247	SAMN03400278
S379V0851	Amplicon	93353	74280	SAMN03400279
S380V0821	Amplicon	103861	87173	SAMN03400282
S383V0880	Amplicon	95136	77196	SAMN03400283
S399V0872	Amplicon	103317	70611	SAMN03400284
S402V0907	Amplicon	107391	86077	SAMN03400285
S405V0882	Amplicon	79536	69498	SAMN03400286
S420V0936	Amplicon	59114	50136	SAMN03400287

Sample Name	Library Type	Number of Reads	Number assigned to OTUs	Accession
S422V0962	Amplicon	119535	97873	SAMN03400288
S427V0938	Amplicon	92832	76970	SAMN03400289
S430V0965	Amplicon	114767	92169	SAMN03400290
S432V0981	Amplicon	172782	135288	SAMN03400291
S441V0932	Amplicon	43974	34082	SAMN03400292
S454C_V3	Amplicon	87209	73419	SAMN03400293
S460V0971	Amplicon	64456	54435	SAMN03400295
S464V1012	Amplicon	135664	105963	SAMN03400296
S466V1010	Amplicon	109782	95810	SAMN03400297
S467V1022	Amplicon	135436	123901	SAMN03400298
S473V1003	Amplicon	97475	77190	SAMN03400299
S474V0998	Amplicon	121534	101809	SAMN03400300
S482V1048	Amplicon	97152	78657	SAMN03400301
S498V1071	Amplicon	153805	130014	SAMN03400302
S501V1097	Amplicon	108341	92452	SAMN03400303
S502V1062	Amplicon	45150	36842	SAMN03400304
S504V1109	Amplicon	39373	33847	SAMN03400305
S511V1126	Amplicon	63006	49369	SAMN03400306
S512V1105	Amplicon	148517	120321	SAMN03400307
S518V1086	Amplicon	104062	84879	SAMN03400308
S520V1118	Amplicon	86289	71795	SAMN03400309
S524V1120	Amplicon	104795	91677	SAMN03400310
S538V1164	Amplicon	91380	79097	SAMN03400311
454C_SG	Shotgun	10176539 (total) 14931 (16S)	11765	SAMN03400294
Tickhill				
TKAC	Amplicon	101204	85897	SAMN03456835
TKDC	Amplicon	104805	59735	SAMN03456836
TKEC	Amplicon	105726	36879	SAMN03456837
TKFC	Amplicon	130710	76605	SAMN03456838
Samdzong				
37C_V3	Amplicon	89494	68253	SAMN03400280
37C_SG	Shotgun	9868955 (total) 17179 (16S)	13397	SAMN03400281
St. Helena				
STH218	Amplicon	222	26	SAMN03456820
STH419	Amplicon	616	47	SAMN03456821
STH343	Amplicon	9116	1702	SAMN03456822
STH319	Amplicon	9425	1003	SAMN03456823
STH358	Amplicon	31257	1619	SAMN03456824
STH284	Amplicon	33082	20125	SAMN03456825
STH449	Amplicon	35598	5704	SAMN03456826
STH389	Amplicon	43644	12366	SAMN03456827
STH219	Amplicon	52986	14048	SAMN03456828
STH423	Amplicon	67718	14206	SAMN03456829
STH359	Amplicon	70060	10941	SAMN03456830
STH474	Amplicon	87051	16368	SAMN03456831
STH268	Amplicon	90400	14073	SAMN03456832
STH359	Amplicon	92507	7973	SAMN03456833
STH430	Amplicon	96331	45919	SAMN03456834
Camino del Molino				
214C_V3	Amplicon	114992	90416	SAMN03400213
214C_SG	Shotgun	11384021 (total) 19552 (16S)	15451	SAMN03400214

Sample Name	Library Type	Number of Reads	Number assigned to OTUs	Accession
Anse à la Gourde				
F0335	Amplicon	53417	37935	SAMN03400217
F0349-A	Amplicon	62256	46173	SAMN03400218
F0430	Amplicon	59713	34934	SAMN03400219
F0529	Amplicon	64071	41271	SAMN03400220
F1948C_V3	Amplicon	102603	78941	SAMN03400221
F1948C_SG	Shotgun	11832345 (total)<br>25418 (16S)	20814	SAMN03400222
Lavoutte				
F67-31	Amplicon	58446	38229	SAMN03400223
F67-33	Amplicon	69578	51500	SAMN03400224
F68-04	Amplicon	27481	15412	SAMN03400225
F69-01	Amplicon	66280	53838	SAMN03400226
F69-05	Amplicon	23815	14199	SAMN03400227
Lab Controls				
Benches	Amplicon	5037	4617	SAMN03400212
HandSurfaceSwabs	Amplicon	21959	20368	SAMN03400230
Blanks				
PCRBlank1	Amplicon	31	17	SAMN03400231
PCRBlank2	Amplicon	635	190	SAMN03400232
ExtractionBlank1	Amplicon	726	316	SAMN03400215

Supplementary Table 5 | Accession IDs for samples used as source environments in this study.

Source	Accession	Database/Reference
Oral	SRS050640, SRS042857, SRS044978, SRS013699, SRS013729, SRS013723, SRS016853, SRS016880, SRS016877, SRS013456, SRS013474, SRS013472, SRS017115, SRS017142, SRS017139, SRS016337, SRS016364, SRS016361, SRS011588, SRS011604, SRS011601, SRS016525, SRS016543, SRS016541, SRS018084, SRS018102, SRS018100, SRS018435, SRS018453, SRS018451, SRS017402, SRS017420, SRS017418, SRS021978, SRS021996, SRS021994, SRS017529, SRS017547, SRS017545, SRS020298, SRS020316, SRS020314, SRS021117, SRS021135, SRS021133, SRS020877, SRS020895, SRS020893, SRS020592, SRS020610, SRS020608, SRS022041, SRS022059, SRS022057, SRS023373, SRS023391, SRS023389, SRS045314, SRS053216, SRS047667, SRS047327, SRS050114, SRS057608, SRS055141, SRS054669, SRS042093, SRS021492, SRS021510, SRS021508, SRS022866, SRS022884, SRS022882, SRS015817, SRS015835, SRS015833, SRS051166, SRS044700, SRS054441, SRS014803, SRS014821, SRS014819, SRS015293, SRS015311, SRS015309, SRS019091, SRS015472, SRS015470, SRS015350, SRS015368, SRS015366, SRS019783, SRS016283, SRS016281, SRS018735, SRS018753, SRS018751, SRS022554, SRS022572, SRS022570, SRS011159, SRS011175, SRS011173, SRS042483, SRS052681, SRS048589, SRS011357, SRS011373, SRS011371, SRS024273, SRS024291, SRS024289, SRS011504, SRS011520, SRS011518, SRS011482, SRS011496, SRS011494, SRS011407, SRS011571, SRS011569	Human Microbiome Project, NCBI SRA database http://www.ncbi.nlm.nih.gov/sra
Gut	SRS016630, SRS016916, SRS016954, SRS017455, SRS018133, SRS018250, SRS018872, SRS019381, SRS020350, SRS020755, SRS020926, SRS021175, SRS021241, SRS021364, SRS022414, SRS022546, SRS022735, SRS022798, SRS023545, SRS023734, SRS023971, SRS024625, SRS024682, SRS042290, SRS042628, SRS042843, SRS045350, SRS045627, SRS047561, SRS047967, SRS048008, SRS048853, SRS049911, SRS050141, SRS055137, SRS056255, SRS056620	
Skin	SRS053492, SRS013738, SRS016892, SRS017154, SRS016376, SRS011611, SRS016551, SRS049441, SRS045992, SRS017428, SRS017555, SRS020324, SRS020903, SRS020618, SRS022067, SRS023399, SRS051229, SRS054830, SRS015811, SRS014831, SRS015289, SRS015340, SRS016263, SRS018761, SRS022580, SRS011181, SRS048113, SRS013096, SRS024299, SRS011444, SRS011500, SRS011577	
Soil	AK.19.12a.414608, AK.19.12a.414616, AK.19.15a.414612, AK.19.18a.414617, FL.3.13a.414620, FL.3.14a.414607, FL.3.16a.414618, HI.20.11a.414613, HI.20.11a.414621, UT.15.42.414609, UT.15.42.414610, UT.15.44.414611, UT.15.45.414619	NEON soils EMP, Study ID 808 http://qitta.microbio.me

Supplementary Table 6 | Relative abundance of genera obtained from analysis of single copy marker loci retrieved from shotgun metagenomic data.

Taxon	C214C_SG	S37C_SG	S454C_SG	F1948C_SG
Actinomyces	0.198	0.19	0.223	0.093
Streptococcus	0.179	0.134	0.037	0.167
Lautropia	0.025	0.116	0.051	0.056
Campylobacter	0.108	0.018	0.055	0.061
Bacteroidetes	0.092	0.034	0.045	0.041
Tannerella	0.041	0.009	0.091	0.008
Eikenella	0.004	0.085	0.028	0.068
Eubacterium	0.009	0.004	0.075	0.023
Gemella	0.005	0.012	0.001	0.069
Cardiobacterium	0.004	0.061	0.005	0.039
Neisseria	0.03	0.057	0.025	0.061
Pseudoramibacter	0.001	0	0.04	0.001
Capnocytophaga	0.008	0.037	0.014	0.022
Parvimonas	0.039	0.004	0.005	0.065
Aggregatibacter	0.005	0.03	0.003	0.032
Slackia	0.032	0.001	0.006	0.001
Porphyromonas	0.004	0.002	0.026	0.003
Corynebacterium	0.006	0.02	0.017	0.025
Kingella	0.002	0.02	0.006	0.013
Prevotella	0.02	0.005	0.015	0.019
Selenomonas	0.017	0.007	0.006	0.019
Rothia	0.003	0.015	0.001	0.001
Treponema	0.009	0.003	0.015	0.005
Catonella	0.007	0.014	0.002	0.01
Pyramidobacter	0.014	0.003	0.008	0
Leptotrichia	0.011	0.006	0.004	0.009
Olsenella	0.004	0.001	0.011	0.001
Desulfovibrio	0.005	0.002	0.01	0
Fusobacterium	0.006	0.005	0.003	0.009
Acidovorax	0.001	0.006	0.008	0.005
Centipeda	0.008	0.001	0.001	0.002
Filifactor	0.001	0.001	0.008	0.001
Burkholderia	0	0.007	0.005	0.001
Desulfomicrobium	0.002	0.001	0.007	0
Veillonella	0.001	0.007	0	0.001
Abiotrophia	0.006	0.001	0.003	0.006
Alicycliphilus	0.001	0.003	0.004	0.006
Isoptericola	0.004	0	0.006	0.001
Polaromonas	0	0.006	0.004	0.003
Chlorobium	0	0.005	0	0
Haemophilus	0.002	0.005	0	0.005
Pseudomonas	0.001	0.005	0	0
Ramlibacter	0	0.005	0.005	0.002
Others[a]	0.085	0.052	0.121	0.046

Notes:

In general, there is good taxonomic correlation between the results obtained from the mOTUs pipeline and the QIIME 16S rRNA pipeline for the shotgun metagenomics data (0.72 ± 0.06, genus level).

[a]Genera with relative abundance less than 0.5% are combined in the category ‘Others’.

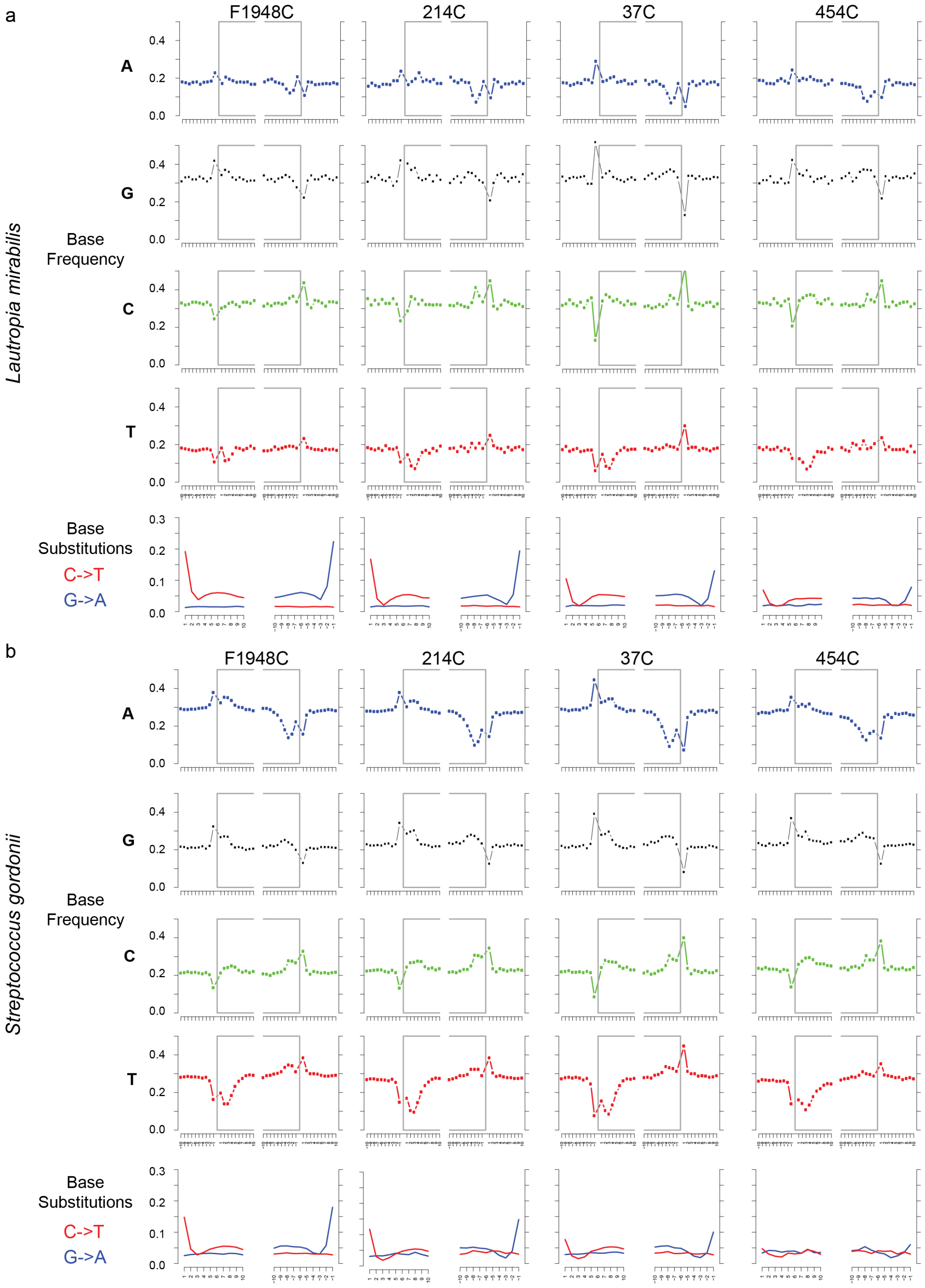

Supplementary Figure 1 | Damage profiles for DNA sequences mapped to two oral bacterial species. Shotgun metagenomics reads were mapped against reference genomes for (**a**) *Streptococcus gordonii* (NC_009785), and (**b**) *Lautropia mirabilis* (GCA_000186425). Fragmentation and misincorporation profiles were generated using the program 'mapDamage' (v2.0)[37]. Each column

represents one ancient dental calculus sample. The first four rows represent nucleotide frequencies for 10 bases upstream and downstream of the 5’ and 3’ regions of the sequenced DNA fragment. The fifth row represents misincorporation curves for (C->T) and (G->A) changes at the 5’ and 3’ regions of the sequenced DNA fragment. Samples are arranged from left to right by decreasing thermal age. All four ancient calculus samples show characteristic patterns of DNA fragmentation, with increased double-stranded breaks after purine (A and G) residues at the 5’ end and before pyramidine residues (T and C) at the 3’ end of the aDNA molecules. Terminal nucleotide misincorporations (5’ C->T; 3’ G->A) were significantly more frequent samples of greater thermal age (Kruskal-Wallis, $p < 0.01$).

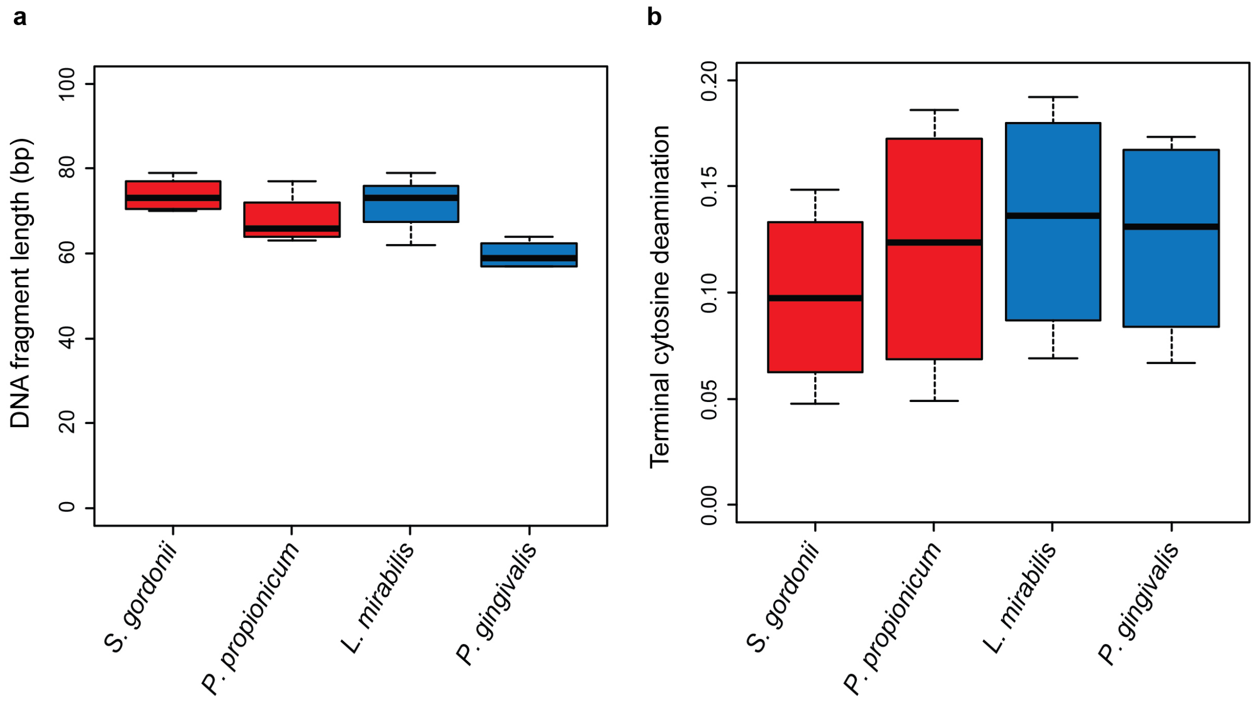

Supplementary Figure 2 | Ancient DNA damage patterns for oral bacteria differing in cell wall composition. Gram-positive taxa (*Streptococcus gordonii* and *Propionibacterium propionicum*) are shown in red; Gram-negative taxa (*Lautropia mirabilis* and *Porphyromonas gingivalis*) are shown in blue. (**a**) Median DNA fragment lengths differ among taxa (Kruskal-Wallis, $p = 0.04$), but not by cell wall composition. (**b**) Terminal cytosine deamination rates also differ among taxa; however the differences are neither significant nor consistent with cell wall composition. For each taxon, box plots were generated from data from four ancient dental calculus samples (454C, 37C, 214C, F1948C). Shotgun metagenomic sequences were mapped against the *S. gordonii* (NC_009785), *P. propionicum* (NC_018142), *L. mirabilis* (GCA_000186425), and *P. gingivalis* (NC_002950) reference genomes.

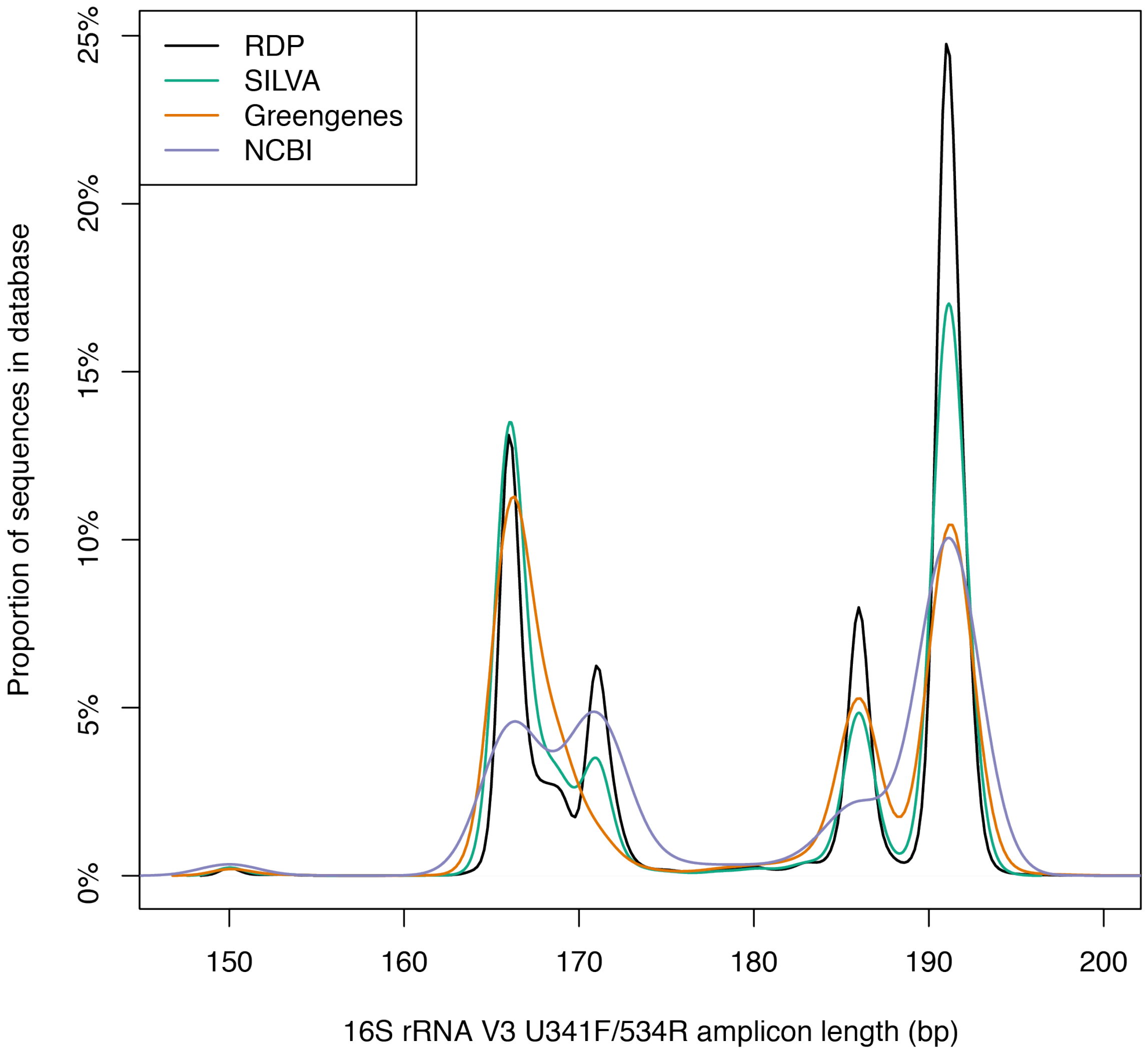

Supplementary Figure 3 | Extensive length polymorphisms in V3 region of the 16S rRNA in multiple databases. Histogram of the predicted amplicon lengths (bp) for the 16S rRNA gene V3 U341F/534R region is shown. Sequence data was obtained from RDP 11.3 (3,019,928 records), NCBI reference genomes (738,633 records; as of 2013-07-30), Greengenes 13 (1,049,116 records) and SILVA SSU 115 (3,194,778 records). A smoothed kernel density line was plotted over each V3 length histogram using R with default density bandwidth parameters. Predicted length polymorphisms are consistent across databases.

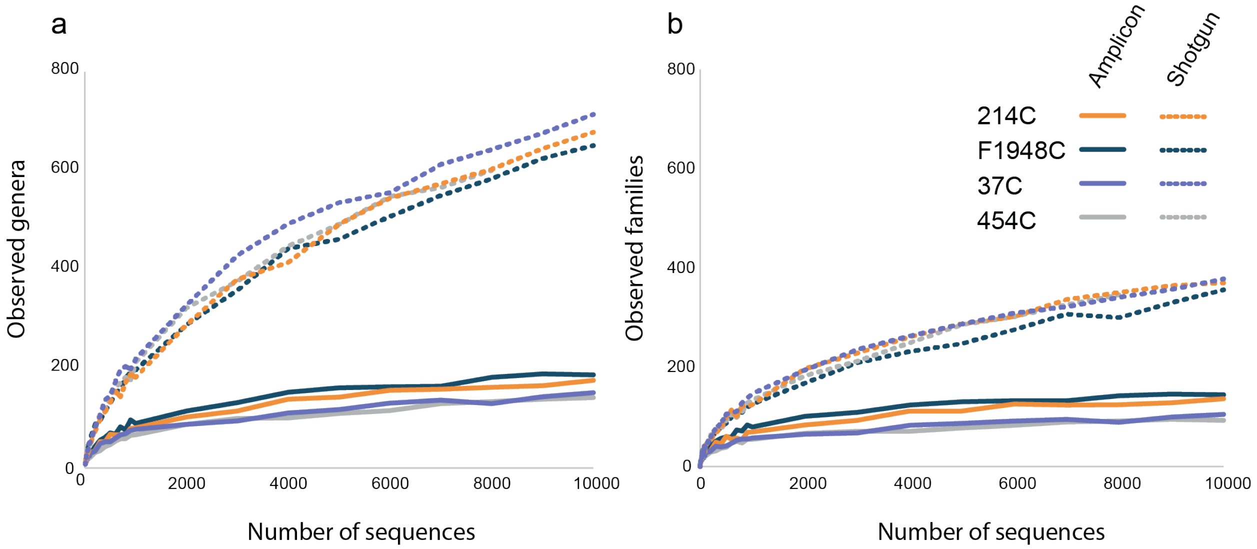

Supplementary Figure 4 | Rarefaction curves for closed reference OTU tables generated from V3 region amplicons, and 16S reads filtered from shotgun metagenome sequencing for the four ancient dental calculus samples. Rarefaction analyses were performed in QIIME[38] from OTU tables summarized at (**a**) Genus level. (**b**) Family level. Both amplicon and shotgun metagenome datasets are approaching plateau, but the shotgun metagenome datasets have increased diversity compared to the amplicon dataset. This is partially caused by the fact that the shotgun metagenome dataset contains a mixture of reads that map along the entire 16S rRNA gene, for which there is variation in OTU assignment information across the different variable regions, as described in Table 3.

References

1 Brooks, S., Suchey, J.M. Skeletal age determination based on the os pubis: a comparison of the Acsádi-Nemeskéri and Suchey-Brooks methods. **5**, 227-238 (Human Evolution).

2 Brothwell, D. R. *Digging up bones : the excavation, treatment, and study of human skeletal remains*. 3rd edn, (Cornell University Press, 1981).

3 Buckberry, J. L. & Chamberlain, A. T. Age estimation from the auricular surface of the ilium: A revised method. *American journal of physical anthropology* **119**, 231-239 (2002).

4 Iscan, M. Y., Loth, S. R. & Wright, R. K. Metamorphosis at the Sternal Rib End - a New Method to Estimate Age at Death in White Males. *American journal of physical anthropology* **65**, 147-156 (1984).

5 Katz, D. & Suchey, J. M. Age determination of the male os pubis. *American journal of physical anthropology* **69**, 427-435 (1986).

6 Lovejoy, C. O., Meindl, R. S., Pryzbeck, T. R. & Mensforth, R. P. Chronological Metamorphosis of the Auricular Surface of the Ilium - a New Method for the Determination of Adult Skeletal Age at Death. *American journal of physical anthropology* **68**, 15-28 (1985).

7 Maat, G. J. Diet and age-at-death determinations from molar attrition. A review related to the low countries. *The Journal of forensic odonto-stomatology* **19**, 18-21 (2001).

8 Meindl, R. S. & Lovejoy, C. O. Ectocranial Suture Closure - a Revised Method for the Determination of Skeletal Age at Death Based on the Lateral-Anterior Sutures. *American journal of physical anthropology* **68**, 57-66 (1985).

9 Todd, T. W. Age Changes in the Pubic Bone. *American journal of physical anthropology* **4**, 1-70 (1921).

10 Anthropologists, W. o. E. Recommendations for Age and Sex Diagnoses of Skeletons. *Journal of human evolution* **9**, 517-& (1980).

11 Ascádi G, N. J. *History of Human Life Span and Mortality*. (Akadémiai Kiadó, 1970).

12 Buikstra, J. E. & Ubelaker, D. H. *Standards for data collection from human skeletal remains. Arkansas Archeological Survey Research Series*. (Arkansas Archeological Survey, 1994).

13 Iordanidis, P. Détermination du sexe par les os du squelette (atlas, axis, clavicule, omoplate, sternum). . *Annales de Médecine Légale* **41**, 280-291 (1961).

14 Phenice, T. W. A newly developed visual method of sexing the os pubis. *American journal of physical anthropology* **30**, 297-301 (1969).

15 Stewart, T. D. *Essentials of Forensic Anthropology Especially as Developed in the United States*. (Charles C. Thomas, 1979).

16 Dobney, K. & Brothwell, D. A Method for Evaluating the Amount of Dental Calculus on Teeth from Archaeological Sites. *Journal of Archaeological Science* **14**, 343-351 (1987).

17 Aldenderfer, M. Variation in mortuary practice on the early Tibetan plateau and the high Himalayas. *Journal of the International Association for Bon Research* **1**, 293-318 (2013).

18 Aldenderfer, M. Final Report: Archaeological research at Choedzom, Upper Mustang, Nepal Grant 8810-10. (National Geographic Society, 2010).

19 Lomba Maurandi, J., Martinez, M. L., Martinez, F. R. & Fernandez, A. A. The collective Chalcolithic burial of Camino del Molino (Caravaca de la Cruz, Murcia, Spain). Methodology and the first results of an exceptional archaeological site. *Trabajos Prehist* **66**, 143-159 (2009).

20 Adler, C. J. *et al.* Sequencing ancient calcified dental plaque shows changes in oral microbiota with dietary shifts of the Neolithic and Industrial revolutions. *Nature genetics* **45**, 450-455 (2013).

21 Luciani, S., Fornaciari, G., Rickards, O., Labarga, C. M. & Rollo, F. Molecular characterization of a pre-Columbian mummy and in situ coprolite. *American journal of physical anthropology* **129**, 620-629 (2006).

22 Ubaldi, M. *et al.* Sequence analysis of bacterial DNA in the colon of an Andean mummy. *American journal of physical anthropology* **107**, 285-295 (1998).

23 Ravel, J. *et al.* Vaginal microbiome of reproductive-age women. *Proceedings of the National Academy of Sciences of the United States of America* **108 Suppl 1**, 4680-4687 (2011).

24 Cano, R. J. *et al.* Sequence analysis of bacterial DNA in the colon and stomach of the Tyrolean Iceman. *American journal of physical anthropology* **112**, 297 (2000).

25 Rollo, F., Luciani, S., Canapa, A. & Marota, I. Analysis of bacterial DNA in skin and muscle of the

Tyrolean iceman offers new insight into the mummification process. *American journal of physical anthropology* **111**, 211-219 (2000).
26 Rollo, F., Luciani, S., Marota, I., Olivieri, C. & Ermini, L. Persistence and decay of the intestinal microbiota's DNA in glacier mummies from the Alps. *Journal of archaeological science* **34**, 1294-1305 (2007).
27 Warinner, C. *et al.* Pathogens and host immunity in the ancient human oral cavity. *Nature genetics* **46**, 336-344 (2014).
28 Baker, G. C., Smith, J. J. & Cowan, D. A. Review and re-analysis of domain-specific 16S primers. *Journal of microbiological methods* **55**, 541-555 (2003).
29 Hansen, M. C., Tolker-Neilson, T., Givskov, M., Molin, S. Biased16S rDNA PCR amplification caused by interference from DNA flanking template region. *FEMS Microbiol. Ecol.* **26**, 141-149 (1998).
30 Tito, R. Y. *et al.* Insights from characterizing extinct human gut microbiomes. *PloS one* **7**, e51146 (2012).
31 Caporaso, J. G. *et al.* Ultra-high-throughput microbial community analysis on the Illumina HiSeq and MiSeq platforms. *The ISME journal* **6**, 1621-1624 (2012).
32 Reysenbach, A. L., Wickham, G. S. & Pace, N. R. Phylogenetic analysis of the hyperthermophilic pink filament community in Octopus Spring, Yellowstone National Park. *Appl Environ Microbiol* **60**, 2113-2119 (1994).
33 Lane, D. J. *et al.* Rapid determination of 16S ribosomal RNA sequences for phylogenetic analyses. *Proceedings of the National Academy of Sciences of the United States of America* **82**, 6955-6959 (1985).
34 Zaura, E., Keijser, B. J. F., Huse, S. M. & Crielaard, W. Defining the healthy "core microbiome" of oral microbial communities. *BMC microbiology* **9**, 259 (2009).
35 Crielaard, W. *et al.* Exploring the oral microbiota of children at various developmental stages of their dentition in the relation to their oral health. *BMC medical genomics* **4**, 22 (2011).
36 Keijser, B. J. F. *et al.* Pyrosequencing analysis of the oral microflora of healthy adults. *Journal of dental research* **87**, 1016-1020 (2008).
37 Jonsson, H., Ginolhac, A., Schubert, M., Johnson, P. L. & Orlando, L. mapDamage2.0: fast approximate Bayesian estimates of ancient DNA damage parameters. *Bioinformatics* **29**, 1682-1684 (2013).
38 Kuczynski, J. *et al.* Using QIIME to analyze 16S rRNA gene sequences from microbial communities. *Current protocols in microbiology* **Chapter 1**, Unit 1E 5 (2012).
